# Empirical Analysis of Hierarchy and Shared Macroscopic Organization in Soccer Leagues

**DOI:** 10.3390/e28090969

**Published:** 2026-08-30

**Authors:** António M. Lopes

**Affiliations:** LAETA/INEGI, Faculty of Engineering, University of Porto, Rua Dr. Roberto Frias, 4200-465 Porto, Portugal; aml@fe.up.pt

**Keywords:** complex systems, non-equilibrium dynamics, Bradley–Terry model, competitive hierarchy, macro-state analysis, sports analytics

## Abstract

This paper presents a coarse-grained framework for analyzing the long-term structural evolution of soccer leagues. This framework draws on ideas from non-equilibrium statistical physics. It combines latent-strength modeling, interaction rules, and large-scale observables to explore the rise of hierarchical organization in competitive leagues. Teams interact based on a Bradley–Terry-type stochastic model. This model relies on teams’ latent competitive strengths, which are derived from match outcomes employing maximum-likelihood estimation. Experiments on 38 soccer leagues around the world show persistent hierarchical organization. This is evident in varied latent strengths, uneven competitive balance, and stable macro-state organization from season to season. Even with differences in history, geography, and competition format, many leagues display surprisingly similar large-scale structural patterns. These findings support the idea of shared principles that guide the evolution of competitive leagues and highlight the value of using a statistical physics perspective to describe how macro-level organization arises from repeated team interactions.

## 1. Introduction

Soccer is the most popular sport in the world [1,2,3]. A typical match is played by two teams for two halves of 45 min each, on a rectangular field with goals at opposite ends. Each team has ten field players and one goalkeeper. The team that scores the most goals wins.

National soccer competitions are usually organized into leagues made up of several teams. Most major European leagues follow a format where all teams play against each other in a double round-robin setup. In this format, each team plays every other team twice, once at home and once away. Teams earn points based on match results: a win gives 3 points, a draw gives 1 point, and a loss gives 0 points. At the end of the season, the team with the most points is declared the champion [4]. Other leagues worldwide use different competition formats. In playoff systems, the regular season determines which teams qualify for a knockout tournament that decides the champion. This format allows lower-ranked teams to win the title with a strong performance in the postseason. Split or championship-stage systems keep the league-table principle but add a second phase where the top teams play more matches against each other. This maintains a competitive title race while emphasizing the importance of consistent performance over the season.

Beyond its role in sports, soccer is a rich example of a complex adaptive system made up of interacting agents that evolve over different spaces and times. Competitive dynamics arise from repeated interactions among players and teams, strategy changes, random fluctuations, and organizational limits. Despite soccer’s social and economic significance, research on league dynamics from the viewpoint of dynamical and complex systems theory is still quite limited.

Previous research has looked into several aspects of soccer dynamics. Couceiro et al. [5] studied predictability and stability using concepts from fractional calculus and entropy. In [6], Araújo et al. introduced dyadic analysis to describe interactions between players, and later extended this approach to examine relationships with teammates and opponents [7,8]. Other work has provided insights into collective tactical behavior [9]. Entropy-based methods have also been applied to measure performance variability and tactical organization [10,11]. Machado and Lopes used multidimensional scaling techniques to explore league competitiveness and team similarity structures [12]. Lopes and Machado also studied league dynamics through information-theoretical and fractional-order approaches [13,14,15]. Sdrolias et al. created a framework based on the Analytic Hierarchy Process (AHP) to assess the organizational and competitive performance of professional clubs [16]. They applied this framework to Panathinaikos in order to find factors behind its competitive decline and aid in strategic repositioning. Jung and Jung looked at count-based and rank-based methods along with Elo and rolling regression analysis in order to predict soccer team strategies and tactical trends based on player composition and performance data from top European leagues [17]. Baratela et al. [18] studied prediction of soccer match outcomes using machine learning models that blend complex passing-network metrics with traditional match statistics, finding that combining these approaches can enhance predictive accuracy and offer deeper insights into team tactics and player interactions. Other studies have examined collective passing behavior [19], predictive ranking dynamics [20], and multilevel network representations of sports systems [21].

A key concept in sports competition is the uncertainty of match outcomes and final rankings. Competitive balance reflects the unpredictability of a league and greatly impacts spectator interest, sponsorship, and economic value [22,23]. Predictable competitions reduce uncertainty and might lower engagement, while too much randomness can disrupt the meaningful structure of competition. Hence, understanding how hierarchy and uncertainty coexist is a crucial issue in sports dynamics [24]. Traditional measures of competitiveness include ratios of success, ranking dispersion, and graph-theoretical indices [15,25,26,27]. Vernon-Carter et al. examined the competitiveness of European soccer leagues by applying SVD entropy to season standings matrices [28]. They assessed the unpredictability of league outcomes and monitored how competitiveness fluctuates among leagues and over time. Cerqueti et al. introduced a new method for ranking soccer teams that incorporates goals scored and conceded [29]. Their method uses Kendall correlation and radar chart-based statistical analysis to improve upon traditional win–draw–loss scoring systems. They applied it to historical data from the Italian Serie A spanning the period from 1930 to 2023. Ficcadenti et al. studied the structure and competitiveness of Italian Serie A soccer [30]. They used a rank-size framework based on the Discrete Generalized Beta Distribution, incorporating clustering and parameter analysis to compare team performance and note changes in league competitiveness over time. Bunker et al. provided an overview of machine learning methods for predicting soccer match outcomes [31]. They compared models and datasets, summarized the best-performing techniques, and highlighted future directions for improvements in feature integration, deeper model comparisons, and better interpretability for practical use in team management.

From the viewpoint of complex systems theory, soccer leagues may be interpreted as open evolving systems, and their macroscopic organization can be studied using concepts inspired by non-equilibrium statistical physics [32,33,34,35]. Throughout this work, the non-equilibrium perspective is employed as a phenomenological coarse-grained framework rather than as a microscopic statistical-mechanical description. Unlike closed systems with fixed parts, leagues change due to ongoing structural shifts caused by promotion and relegation. While the identity of a league remains over time, team memberships change each season as weaker teams drop out and new teams join from lower divisions. This leads to league evolution arising from internal competition and external structural changes.

A significant observation is that league organization evolves. Initially similar teams gradually become different, creating lasting hierarchical structures marked by uneven competition influence and performance. This suggests that a process analogous to symmetry-breaking governs league evolution. Here, the idea of symmetry breaking serves as a useful analogy. It refers to the development of different competitive roles that arise from an initially equal modeling assumption in which no inherent strength differences are defined beforehand. We do not propose a strict spontaneous symmetry-breaking mechanism in the statistical-mechanical sense. In an ideal symmetrical state, teams are nearly interchangeable and match results are very unpredictable; however, repeated competition over time creates differences in performance and stability in rankings, resulting in organized hierarchical states.

In this work, we propose a framework for analyzing the long-term evolution of soccer leagues using concepts inspired by non-equilibrium statistical physics. This framework combines the following elements:

- Latent-strength modeling;
- Stochastic interaction rules based on Bradley–Terry dynamics;
- Inference of hidden competitive structures from match data;
- Macroscopic measures that show entropy, inequality, hierarchy formation, and similarities across leagues.

The main innovation of this work is a unified coarse-grained framework that connects team interactions at the microscopic level with the emergence of league-level competitive organization. The goal of the framework is not to derive microscopic evolution equations, entropy production, or detailed-balance relations, nor to establish universality in the strict statistical-physics sense associated with critical phenomena. Instead, these concepts provide a unifying language for interpreting the emergence and evolution of league-level competitive structure. The framework represents each season by a macroscopic state constructed from observed match outcomes, enabling a geometric analysis of the long-term structural evolution of soccer leagues. By linking latent-strength inference through a Bradley–Terry model with system-level measures such as entropy, inequality, and rank stability, this study goes beyond just predicting matches and analyzing competitive balance to investigate the emergence of competitive hierarchy and self-organization in league dynamics. Additionally, finding similar large-scale patterns in different leagues suggests the existence of common macroscopic organizational principles.

The rest of the paper is organized as follows: Section 2 gives a brief overview of the dataset used in this study; Section 3 presents the proposed coarse-grained dynamical framework for representing the temporal evolution of soccer leagues through season-dependent macro-states; Section 4 defines the macroscopic measures required to create the macro-state vector that describes league dynamics; Section 5 shares and discusses the main results through both within-league and cross-league analyses; finally, Section 6 summarizes the key findings and outlines the main conclusions.

## 2. Description of the Dataset

The dataset consists of match-level records from 38 soccer leagues spanning several continents and competitive structures.

For each league, all available seasons up to the 2025–2026 season (or 2025, for those played during single years) were collected from the public repository provided by https://football-data.co.uk/ (accessed on 26 August 2026). Each league was stored as a single file containing all corresponding data. The temporal coverage varied across leagues depending on data availability and historical continuity.

Each match record was filtered so that League division, match date, time of match kick off, home team, away team, full-time home team goals, full-time away team goals, full-time result, and season data were retained while other available raw information was discarded.

The resulting dataset defines a hierarchical structure of the following form: league–season–match, where leagues represent evolving competitive systems, seasons correspond to discrete temporal realizations, and matches constitute microscopic interaction events between teams.

The leagues exhibit heterogeneous characteristics, including different numbers of participating teams, distinct temporal depths, and varying degrees of membership turnover due to promotion, relegation, playoffs, or split-stage formats. Consequently, the dataset naturally represents an ensemble of open competitive systems with time-dependent state-spaces. Table 1 summarizes a few simple statistics of the 38 soccer leagues.

## 3. Dynamical System Modeling

Within the proposed coarse-grained framework, each season is represented by a macroscopic state inferred from match outcomes. The sequence of seasonal states defines a trajectory in macro-state space, providing a geometric description of the long-term structural evolution of the league. Unlike closed equilibrium systems, leagues dynamics are continuously shaped by competitive interactions, strategic adaptation, stochastic fluctuations, and seasonal changes in membership.

The global dynamical system corresponds to the league observed over multiple seasons. Therefore, the league is interpreted as a sequence of season-dependent competitive subsystems:

(1) $$L={\left\{S_{t}\right\}}_{t=1}^{T},$$

where L denotes the full league dynamical system, $S_{t}$ denotes the subsystem associated with season *t*, and *T* denotes the total number of observed seasons. This notation defines how time is organized in the observed system. It should not be seen as a small-scale evolution operator connecting consecutive seasons. Instead, each seasonal subsystem is derived independently from the observed match outcomes. The resulting sequence of macro-states gives a broad description of how the league has changed over time.

Thus, the league defines the macroscopic evolving system, while each season corresponds to a mesoscopic realization of the competitive environment with partially persistent membership, since team composition may change across seasons.

Each season subsystem $S_{t}$ is defined as a structured object composed of interacting components:

(2) $$S_{t}=\left(V_{t},E_{t},Y_{t}\right),$$

where $V_{t}$ is the set of teams participating in season *t*, $E_{t}$ is the set of matches (pairwise interactions), and $Y_{t}$ denotes the set of observed outcomes associated with matches.

The set of teams is given by

(3) $$V_{t}=\left\{i_{1}^{\left(t\right)},i_{2}^{\left(t\right)},\ldots ,i_{N_{t}}^{\left(t\right)}\right\},$$

where $N_{t}$ denotes the number of teams in season *t*.

The teams constitute the interacting agents of the dynamical system. Each team $i\in V_{t}$ is characterized by a latent competitive strength:

(4) $$s_{i}\left(t\right)\in R$$

representing its underlying competitive ability during season *t*. The collection of all strengths in season *t* is denoted by

(5) $$s_{t}={\left\{s_{i}\left(t\right)\right\}}_{i\in V_{t}}.$$

Therefore, the league is interpreted as a time-evolving latent competitive field defined over a changing set of interacting agents.

At the microscopic level, the fundamental interaction event is the match between two teams. Each match $m\in E_{t}$ involves a pair of teams (i,j) and generates an observed outcome $y_{ij}\left(t\right)\in Y_{t}$. The collection of all matches defines a temporal interaction evolving network. League structure evolves through the combined effects of microscopic competitive interactions generated by matches, the persistence and evolution of latent team strengths, and external membership turnover. Since the number and identity of teams vary with time, the dimensionality of the system is itself time-dependent. Therefore, the league behaves as an open dynamical structure rather than a closed equilibrium system.

### 3.1. Multi-Scale Dynamical Structure

League evolution is governed by three coupled temporal layers operating at different scales.

#### 3.1.1. Within-Season Dynamics

Over a season *t*, teams interact through matches within a fixed competitive environment defined by the subsystem $S_{t}=\left(V_{t},E_{t},Y_{t}\right)$. The number of teams $N_{t}=\left|V_{t}\right|$ is fixed within each season, although it may vary across seasons.

Each team $i\in V_{t}$ is characterized by a latent competitive strength $s_{i}\left(t\right)\in R$, representing its real performance level during season *t*. This latent variable is not directly observable and must be inferred from match outcomes.

In the perfect symmetric regime, all teams are statistically equivalent:

(6) $$s_{i}\left(t\right)=s_{j}\left(t\right),\;\forall i,j\in V_{t}.$$

In this limit, match outcomes are totally uncertain and the system exhibits no hierarchical structure. Deviations from this symmetric state generate persistent asymmetries in latent strengths, leading to the emergence of competitive hierarchy. This process is interpreted as symmetry breaking within the season subsystem.

To capture both persistent and transient effects, we decompose latent strength as

(7) $$s_{i}\left(t\right)=\mu_{i}+\delta_{i}\left(t\right),$$

where $\mu_{i}$ represents the long-term intrinsic ability of team *i* and $\delta_{i}\left(t\right)$ represents seasonal fluctuations and short-term variability. This decomposition separates stable hierarchical structure from temporal perturbations.

Match outcomes are considered to arise from stochastic pairwise interactions governed by latent strength differences. For a match between teams $i,j\in V_{t}$, we define

(8) $$\Delta s_{ij}\left(t\right)=s_{i}\left(t\right)-s_{j}\left(t\right)$$

and model the probability of team *i* defeating team *j* using a Bradley–Terry logistic interaction law:

(9) $$P\left(i\;\mathrm{defeats}\;j\right)=\frac{1}{1+e^{-\Delta s_{ij}\left(t\right)}}.$$

This interaction rule defines the microscopic stochastic dynamics generating observed match outcomes.

Latent strengths are inferred from observed match results independently for each season *t*. Given the set of matches indexed by $m\in E_{t}$, the seasonal log-likelihood is given by

(10) $$L_{t}\left(s_{t}\right)=\sum_{m\in E_{t}}\left[y_{m}\log p_{m}\left(t\right)+\left(1-y_{m}\right)\log\left(1-p_{m}\left(t\right)\right)\right],$$

where

(11) $$p_{m}\left(t\right)=\frac{1}{1+\exp\;\left[-\left(s_{i(m)}\left(t\right)-s_{j(m)}\left(t\right)\right)\right]}.$$

Match outcomes are encoded as $y_{m}=1$ for a win by team i(m), $y_{m}=0$ for a win by team j(m), and $y_{m}=\frac{1}{2}$ for a draw, where i(m),j(m) denote the teams participating in match *m*, such that $y_{m}\equiv y_{i(m),j(m)}$. As such, drawn matches are included as fractional outcomes within a binary Bradley–Terry likelihood. This approach ensures that the impact of drawn matches on the estimation of latent strengths is retained without the need to introduce a further draw parameter. The aim of the current study is to infer the latent strengths at the season level rather than to carry out a detailed probabilistic modeling of the match outcomes.

The Bradley–Terry framework was selected because it provides a probabilistic latent strength model that is directly inferred from pairwise match outcomes through maximum-likelihood estimation. Unlike sequential rating systems such as Elo or Glicko [36,37], which are primarily designed for online rating updates and prediction, the Bradley–Terry model estimates a consistent set of latent strengths for an entire season. This makes it particularly suitable for constructing season-level macroscopic observables and for comparing the structural organization of different leagues. The proposed framework is independent of the specific latent-strength inference model. Any method capable of producing season-level latent strengths could in principle be used in place of Bradley–Terry, although the resulting quantitative values of the macroscopic observables may differ.

To ensure identification and numerical stability of the inferred latent strengths ${\hat{s}}_{i}\left(t\right)$, we introduce an $L^{2}$ regularization term corresponding to a Gaussian prior on the latent team strengths. The estimation problem then becomes

(12) $${\hat{s}}_{t}={\left\{{\hat{s}}_{i}\left(t\right)\right\}}_{i\in V_{t}}=\arg\max_{s_{t}}\left[L_{t}\left(s_{t}\right)-\lambda \sum_{i\in V_{t}}s_{i}{\left(t\right)}^{2}\right],$$

where λ>0 is a regularization parameter controlling the magnitude of penalization. Throughout this paper, the regularization parameter is fixed at λ=0.1, which provides numerically stable estimates.

Regularization removes the translational degeneracy in the Bradley–Terry likelihood, stabilizes inference in sparse or poorly informative seasons, and suppresses bad solutions in which latent strengths become weakly constrained. Therefore, the inferred strengths should be regarded as maximum a posteriori estimates under a Gaussian prior that is centered at zero.

#### 3.1.2. Cross-Season Persistence

For teams that remain in the league across multiple seasons, inferred competitive strength may exhibit temporal persistence associated with factors such as tactical adaptation, financial investment, managerial changes, player transfers, and stochastic fluctuations.

To characterize long-term organization, we represent the inter-season evolution of inferred team strengths through the following relation:

(13) $${\hat{s}}_{i}\left(t+1\right)={\hat{s}}_{i}\left(t\right)+\Gamma_{i}\left(t\right)+\eta_{i}\left(t\right)$$

where $\Gamma_{i}\left(t\right)$ represents a systematic slow component estimated through temporal smoothing of the sequence ${\left\{{\hat{s}}_{i}\left(t\right)\right\}}_{i\in V_{t}}$, and $\eta_{i}\left(t\right)$ is a zero-mean residual term capturing unpredictable fluctuations. This relation is intended as a reconstructed effective dynamic based on inferred seasonal states rather than as a generative dynamic model for use during inference. Each subsystem $S_{t}$ acts as an independent example of the competitive dynamics defined over a season-dependent group of teams. We perform independent seasonal estimation because of changes in league composition. Despite estimating the strengths separately for each season, the resulting sequence of inferred states ${\left\{\hat{s}\left(t\right)\right\}}_{i\in V_{t}}$ often shows significant temporal persistence. Teams with high inferred strengths usually remain competitive leaders across several seasons. This suggests a long-term structural organization and a path dependence in the league hierarchy.

It is important to note that a fully generative dynamic formulation would directly include temporal dependence in the inference process. In that case, latent strengths would change according to a random state-transition rule, and temporal persistence would be a key part of the inference model, not just an observed property reconstructed from estimated seasonal states.

#### 3.1.3. Membership Turnover Dynamics

League composition changes across seasons because some teams exit the league while new teams enter through various competition-specific mechanisms; consequently, in general, $V_{t}\neq V_{t+1}$.

League evolution results from the combined action of internal competitive interactions between teams within a season, temporal persistence of competitive structure across seasons, and external structural perturbations generated by team entries and exits. Since the model does not explicitly consider lower divisions or qualification/relegation mechanisms, transitions between seasons are treated as external perturbations acting on the observed league subsystem. In this sense, the league behaves as an open dynamical system exchanging agents with an external competitive environment.

Membership turnover continuously changes both the small-scale and large-scale structure of the league. On a small scale, new memberships and departures affect the teams that interact and the distribution of hidden strengths. On a large scale, turnover influences observable changes. As a result, each season represents a partial persistence realization of a competitive structure that evolves from the interaction between internal competitive forces and external membership changes.

## 4. Macroscopic Observables

The inferred latent strengths define the microscopic competitive state of each season subsystem. From these inferred states and match results, we create macro observables that describe the overall organization of the league. Macro observables serve as indicators of soccer’s competitive structure. While individual match outcomes show local interactions between teams, these macro observables reflect broader features such as competitive balance, hierarchy formation, structural inequality, and long-term organization models.

### 4.1. Variance

Variance is a measure of strength dispersion, characterizing the global separation between teams through the variance of inferred latent strengths:

(14) $$\sigma_{t}^{2}=\frac{1}{N_{t}}\sum_{i\in V_{t}}{\left({\hat{s}}_{i}\left(t\right)-{\bar{s}}_{t}\right)}^{2}$$

where

$${\bar{s}}_{t}=\frac{1}{N_{t}}\sum_{i\in V_{t}}{\hat{s}}_{i}\left(t\right)$$

is the mean inferred strength.

An increase in $\sigma_{t}^{2}$ corresponds to growing competitive stratification between strong and weak teams, providing a macroscopic measure of hierarchical differentiation within the league.

### 4.2. Gini Coefficient

The Bradley–Terry latent strengths are defined on an arbitrary additive scale, and may be negative. To avoid inconsistencies, inequality is computed from the exponentiated strengths $w_{i}\left(t\right)=\exp\;\left({\hat{s}}_{i}\left(t\right)\right)$. This transformation is the natural parameterization of the Bradley–Terry model, where match probabilities depend on ratios of positive competitive weights rather than on latent strengths directly. This guarantees positivity, preserves the ordering of teams, and is invariant under additive shifts of the estimates of latent strength. By normalizing the resulting weights, we define a probability distribution over competitive strength, which allows us to use of Shannon entropy as a measure of the structural dispersion of competitive ability within the league.

The competitive inequality is then quantified by the Gini coefficient:

(15) $$G_{t}=\frac{\sum_{i\in V_{t}}\sum_{j\in V_{t}}\left|w_{i}\left(t\right)-w_{j}\left(t\right)\right|}{2N_{t}^{2}{\bar{w}}_{t}}$$

where ${\bar{w}}_{t}=\frac{1}{N_{t}}\sum_{i\in V_{t}}w_{i}\left(t\right)$.

Low values of $G_{t}$ correspond to relatively homogeneous competitive structures, whereas large values indicate increasing concentration of competitive ability and stronger hierarchical organization.

### 4.3. Outcome Entropy

The outcome entropy quantifies the uncertainty of the binary predictive distribution generated by the Bradley–Terry model.

For each match $m\in E_{t}$, let

(16) $$p_{m}\left(t\right)=\frac{1}{1+\exp\;\left[-\left({\hat{s}}_{i(m)}\left(t\right)-{\hat{s}}_{j(m)}\left(t\right)\right)\right]},$$

where $p_{m}\left(t\right)$ denotes the model-implied probability of team i(m) defeating team j(m). The outcome entropy is defined as the average predictive entropy over matches:

(17) $$H_{t}^{out}=\frac{1}{|E_{t}|}\sum_{m\in E_{t}}\left[-p_{m}\left(t\right)\log p_{m}\left(t\right)-\left(1-p_{m}\left(t\right)\right)\log\left(1-p_{m}\left(t\right)\right)\right]$$

where $p_{m}\left(t\right)$ is computed from the Bradley–Terry likelihood model $L_{t}\left({\hat{s}}_{t}\right)$. High entropy corresponds to balanced and unpredictable competition, while low entropy indicates increasing structural organization and reduced competitive uncertainty. In the ideal symmetric regime, outcome probabilities are approximately uniform, producing maximal entropy. Deviations from this regime reflect progressive competitive differentiation and hierarchy formation.

### 4.4. Structural Entropy

Structural entropy characterizes the organization of the latent competitive field. We define a normalized softmax (Gibbs-like) distribution over teams,

(18) $$p_{i}\left(t\right)=\frac{\exp({\hat{s}}_{i}\left(t\right))}{\sum_{j\in V_{t}}\exp\left({\hat{s}}_{j}\left(t\right)\right)}.$$

Then, the structural entropy is defined as

(19) $$H_{t}^{struct}=-\sum_{i\in V_{t}}p_{i}\left(t\right)\log p_{i}\left(t\right).$$

This macroscopic variable measures the effective concentration of competitive advantage. Low structural entropy corresponds to highly hierarchical leagues dominated by a small subset of teams, while high structural entropy corresponds to more homogeneous competitive structures.

### 4.5. Concentration Ratio of the Top Teams

Let the transformed strengths be ordered as $w_{\left(1\right)}\left(t\right)\geq w_{\left(2\right)}\left(t\right)\geq \cdots \geq w_{\left(N_{t}\right)}\left(t\right)$. The concentration ratio of the top *k* teams is defined as

(20) $$CR_{t}^{k}=\frac{\sum_{i=1}^{k}w_{\left(i\right)}\left(t\right)}{\sum_{i\in V_{t}}w_{i}\left(t\right)}.$$

The concentration ratio measures the fraction of the total competitive strength concentrated in the strongest teams. High values indicate dominance by a small elite, whereas lower values correspond to a more evenly distributed competitive structure.

### 4.6. Empirical Homefield Advantage

The empirical homefield advantage is defined as the normalized excess of home wins over away wins:

(21) $$HA_{t}=\frac{N_{H}\left(t\right)-N_{A}\left(t\right)}{|E_{t}|}$$

where $N_{H}\left(t\right)$ and $N_{A}\left(t\right)$ denote the number of home wins and away wins in season *t*, respectively.

Positive values indicate a systematic advantage for home teams, while values close to zero correspond to symmetric or weakly biased competitive environments. This observable captures external forcing effects that are not explained by latent team strengths alone.

### 4.7. Macro State-Space

To characterize the large-scale dynamical organization of soccer leagues, we represent each season as a point in a multidimensional macro state-space constructed from collective observables derived from the inferred latent strength field. Rather than analyzing isolated observables independently, this framework captures the joint organization of hierarchy, uncertainty, concentration, and competitive asymmetry as components of a unified dynamical state. The temporal evolution of a league defines a trajectory through this space.

For a league L and season *t*, the macro state vector is defined as

(22) $$X_{L,t}=\left(\sigma_{t}^{2},G_{t},H_{t}^{out},H_{t}^{struct},CR_{t}^{k},HA_{t}\right)\in R^{6}.$$

This construction enables direct comparison between leagues with different microscopic characteristics. If distinct leagues evolve along similar trajectories or occupy similar regions of state-space, then this suggests the existence of shared macroscopic organizational principles.

## 5. Experimental Results

The experimental analysis is divided into two parts. First, we look at the internal temporal patterns of individual leagues by examining the development of hidden strengths and observable data derived from the inferred hidden strength field. We analyze the observable data separately and represent them within a 6D macro-state vector. This analysis describes the internal dynamic structure of each league. Second, we perform a cross-league comparative analysis by embedding all leagues into a common macro-state space. This enables the study of large-scale similarities and differences between leagues with distinct geographic, historical, financial, and organizational characteristics. In particular, we examine whether normalized league trajectories exhibit common geometric organization, clustering behavior, low-dimensional structure, and signatures of shared macroscopic organization. Together, these two levels of analysis provide both a microscopic-to-macroscopic characterization of individual leagues and a global comparative framework for identifying common dynamical principles across soccer leagues.

### 5.1. League-Level Analysis

The inferred latent strengths ${\hat{s}}_{t}$ form the microscopic state of the league, while the macroscopic observables $\left\{\sigma_{t}^{2},G_{t},H_{t}^{out},H_{t}^{struct},CR_{t}^{k},HA_{t}\right\}$, t=1,⋯,T describe collective properties emerging from the interaction between teams over repeated competitive seasons. Despite the study analyzing all 38 leagues, detailed results are illustrated only for {England_Premier} and {Japan_JLeague} as two representative leagues, in order to avoid overwhelming the paper with somewhat repetitive descriptions.

#### 5.1.1. Latent Competitive Field

The first conducted analysis addresses the evolution of inferred latent strengths through time using strength trajectories, seasonal heatmaps, persistence analysis, and quantile–quantile (QQ) plots.

Strength trajectories and heatmaps provide a direct visualization of the temporal evolution of the latent competitive field, allowing for identification of stable elite teams, periods of restructuring, and long-term competitive stability. Persistence measures are related to the temporal memory of the league hierarchy, indicating whether competitive order remains stable or undergoes reorganization. QQ-plots compare the shape of latent strength distributions across seasons. They can show whether the underlying competitive structure is statistically stable or whether it shows systematic deformation. This may include the appearance of heavy tails, concentration effects, or symmetrical breaking of hierarchy.

Latent strengths were first standardized independently within each season using *z*-score normalization:

(23) $${\tilde{s}}_{i}\left(t\right)=\frac{{\hat{s}}_{i}\left(t\right)-\bar{\hat{s}}\left(t\right)}{\sigma (t)}$$

where ${\tilde{s}}_{i}\left(t\right)$ denotes the normalized latent strength of team *i*, $\bar{\hat{s}}\left(t\right)$ is the mean strength, and σ(t) is the standard deviation of strengths in season *t*. This method removes the arbitrary translation and scale issues that come with Bradley–Terry estimation. This ensures that strengths can be directly compared as relative deviations from the league average within each season.

**Figure 1:**
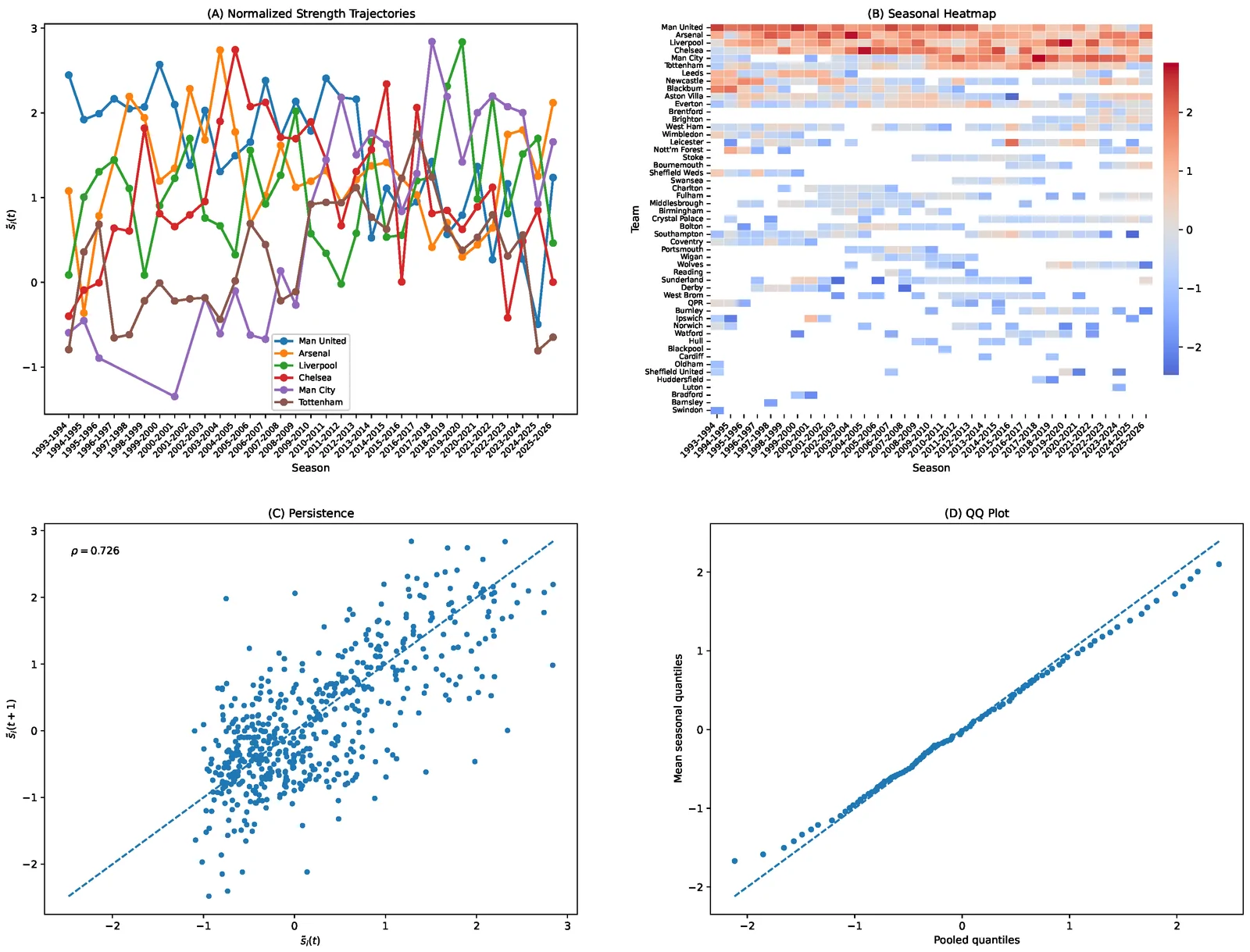
Evolution of normalized latent strength of {England_Premier}: (**A**) trajectories ${\tilde{s}}_{i}\left(t\right)$ of the top six teams; (**B**) heatmap of ${\tilde{s}}_{i}\left(t\right)$; (**C**) persistence ${\tilde{s}}_{i}\left(t+1\right)$ versus ${\tilde{s}}_{i}\left(t\right)$; (**D**) QQ-plot.

**Figure 2:**
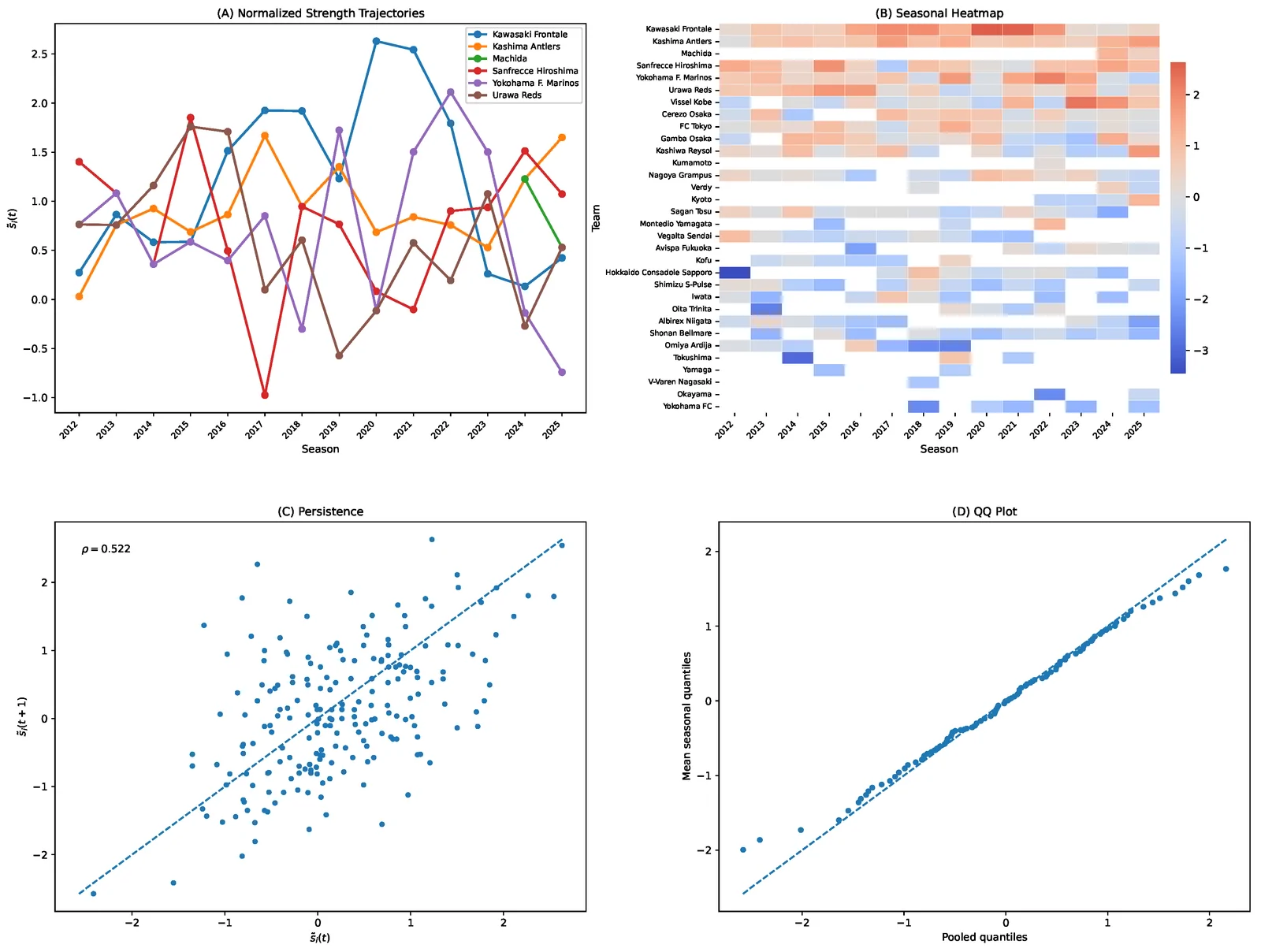
Evolution of normalized latent strength of {Japan_JLeague}: (**A**) trajectories ${\tilde{s}}_{i}\left(t\right)$ of the top six teams; (**B**) heatmap of ${\tilde{s}}_{i}\left(t\right)$; (**C**) persistence ${\tilde{s}}_{i}\left(t+1\right)$ versus ${\tilde{s}}_{i}\left(t\right)$; (**D**) QQ-plot.

Figure 1 and Figure 2 illustrate the seasonal evolution of ${\tilde{s}}_{i}\left(t\right)$ of the top six teams of leagues {England_Premier} and {Japan_JLeague}. The teams were ranked according to their time-averaged normalized latent strength across all observed seasons:

(24) $${\bar{\tilde{s}}}_{i}=\frac{1}{T_{i}}\sum_{t}{\tilde{s}}_{i}\left(t\right)$$

where $T_{i}$ is the total number of seasons in which team *i* has competed. The figures additionally present the heatmap of ${\tilde{s}}_{i}\left(t\right)$, the persistence relation between ${\tilde{s}}_{i}\left(t+1\right)$ and ${\tilde{s}}_{i}\left(t\right)$, and the QQ-plot comparing the quantiles of the pooled normalized latent-strength distribution across all seasons with the mean seasonal quantiles.

For each league, the strength patterns of the top six teams show changes over time and some overlap. This means that competitive dominance develops over time instead of being completely fixed. In {England_Premier}, it seems that Man City and Tottenham joined the group of top teams starting in 2009–2010, with both showing more consistency since then. The seasonal heatmaps back up these patterns by showing stable yet changing rankings across different seasons. In {Japan_JLeague}, there was a period of increased volatility from 2017 to 2022. Persistence analysis shows moderate continuity in team strengths from season to season. {England_Premier} had a stronger persistence score of ρ=0.726, compared to ρ=0.522 for {Japan_JLeague}. This suggests that there is more team movement in {Japan_JLeague}. Additionally, the QQ-plots remain close to the diagonal reference line. This shows that the structure of the normalized strengths stays relatively stable over time for both leagues.

#### 5.1.2. Distributional Geometry of Competitive Strengths

**Figure 3:**
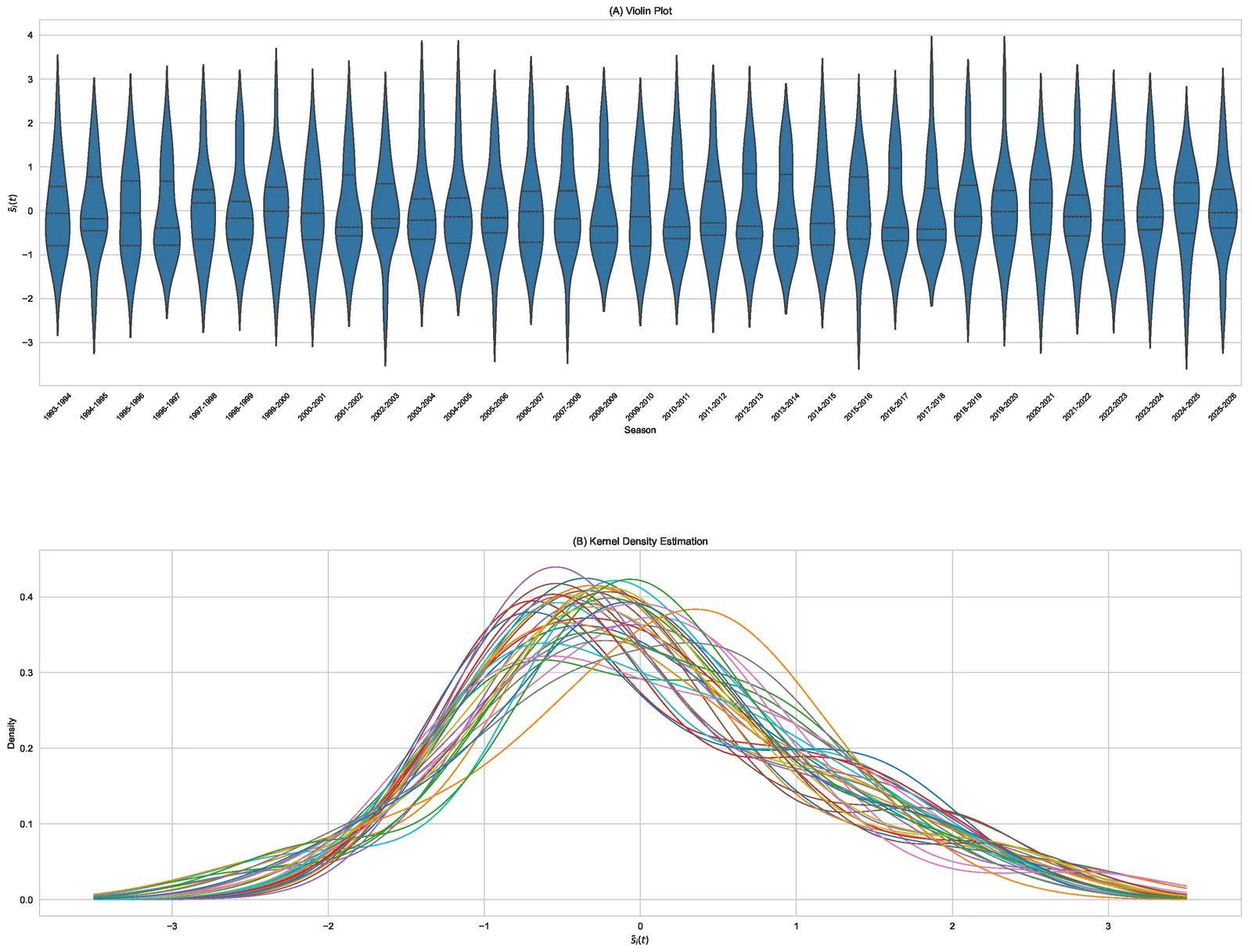
Distributional structure of normalized latent strengths of {England_Premier}: (**A**) violin plots of ${\tilde{s}}_{i}\left(t\right)$ and (**B**) KDE of ${\tilde{s}}_{i}\left(t\right)$.

**Figure 4:**
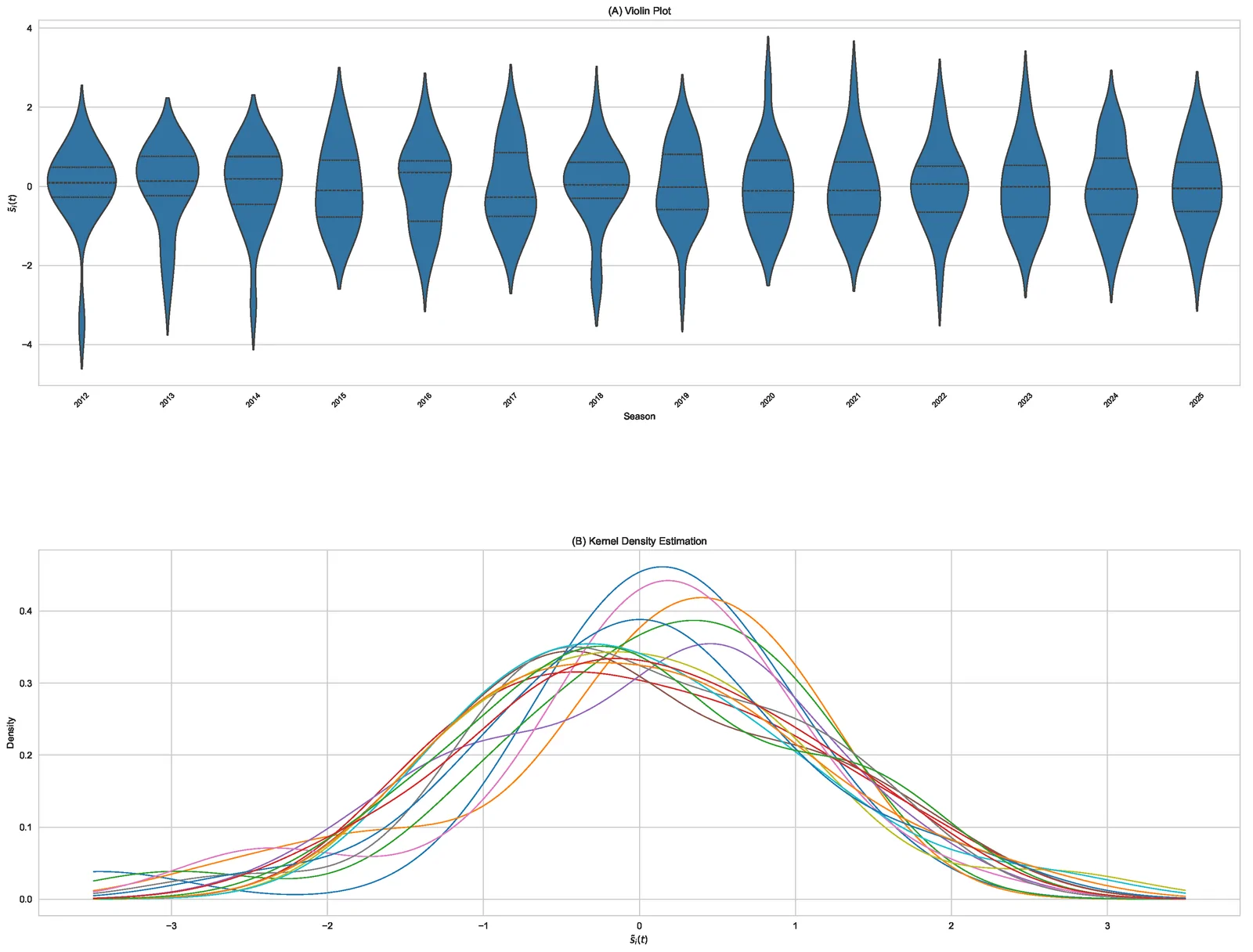
Distributional structure of normalized latent strengths of {Japan_JLeague}: (**A**) violin plots of ${\tilde{s}}_{i}\left(t\right)$ and (**B**) KDE of ${\tilde{s}}_{i}\left(t\right)$.

Distributional structure of latent strengths are assessed by means of violin plots and kernel density estimates (KDEs), as depicted in Figure 3 and Figure 4 for {England_Premier} and {Japan_JLeague}, respectively. These representations illustrate the geometric structure of the competitive environment, including possible multimodality, concentration of competitive power, and distributional asymmetry.

For the two leagues, the normalized latent-strength distributions remain broadly stable across seasons, pointing to a persistent underlying competitive structure. However, several seasons exhibit noticeable deviations from this pattern, presenting increased skewness and heavier tails. This is seen in {England_Premier} during 2004–2005 (in this season, Chelsea won the title with a then-record 95 points and ended an Arsenal’s period of supremacy) as well as in 2017–2018, 2018–2019, and 2019–2020 (seasons marked by historically high point totals by the leading teams, corresponding to an unusually pronounced competitive hierarchy). For {Japan_JLeague}, the effect is pronounced in 2020 and 2021, which may have been caused by disruptions due to the COVID-19 pandemic such as schedule interruptions, attendance restrictions, and temporary changes to promotion and relegation rules. The mentioned observations may be interpreted as episodic deviations in competitive balance and the emergence of more extreme team performance disparities.

#### 5.1.3. Hierarchical Organization

We next look into the development of hierarchy using rank plots, variance of strengths, and the Gini coefficient. Rank plots show the ordered structure produced by the latent strength field. They reveal whether the competitive organization follows certain rank-size relationships. Consistency of the rank structure over time shows stability of the competitive hierarchy; in contrast, large fluctuations suggest structural instability and greater competitive balance. For each season, teams are ranked based on their inferred latent strength. The rank of team *i* in season *t* is defined as follows:

(25) $$r_{i}\left(t\right)=\mathrm{rank}\left({\tilde{s}}_{i}\left(t\right)\right).$$

The variance of strengths quantifies the overall amplitude of competitive stratification. The Gini coefficient measures inequality in the distribution of competitive power.

**Figure 5:**
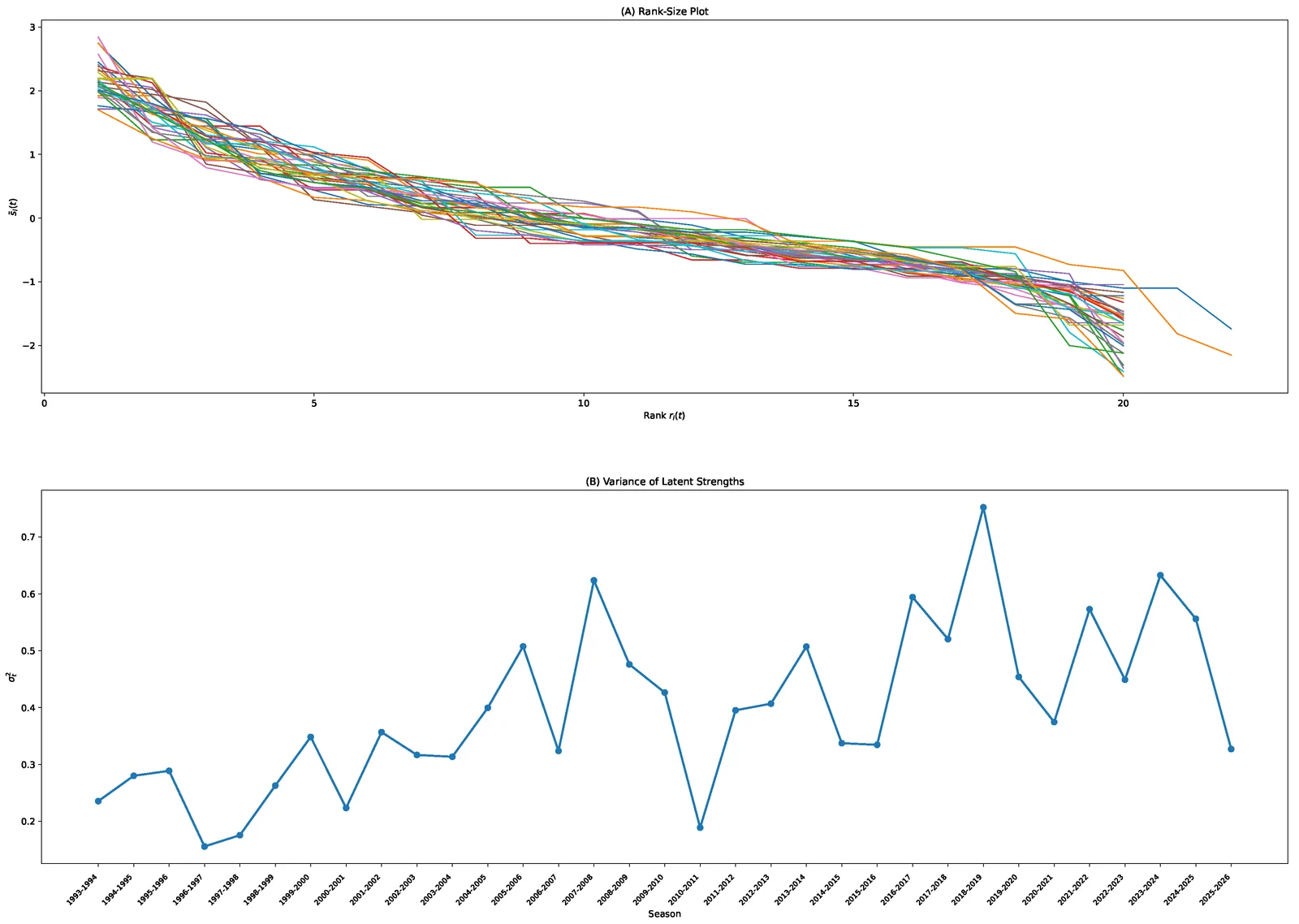

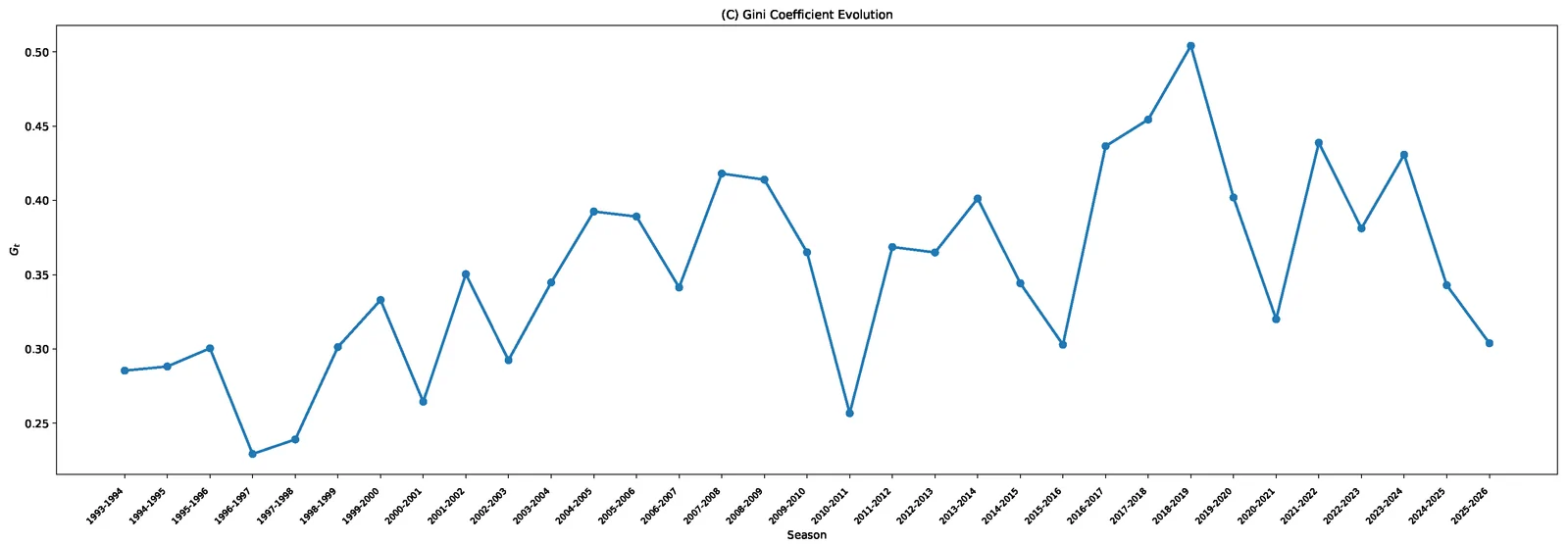
Hierarchical organization of {England_Premier}: (**A**) rank-size visualizations of standardized latent strengths; (**B**) variance of non-standardized latent strengths; and (**C**) Gini coefficient of non-standardized latent strengths.

**Figure 6:**
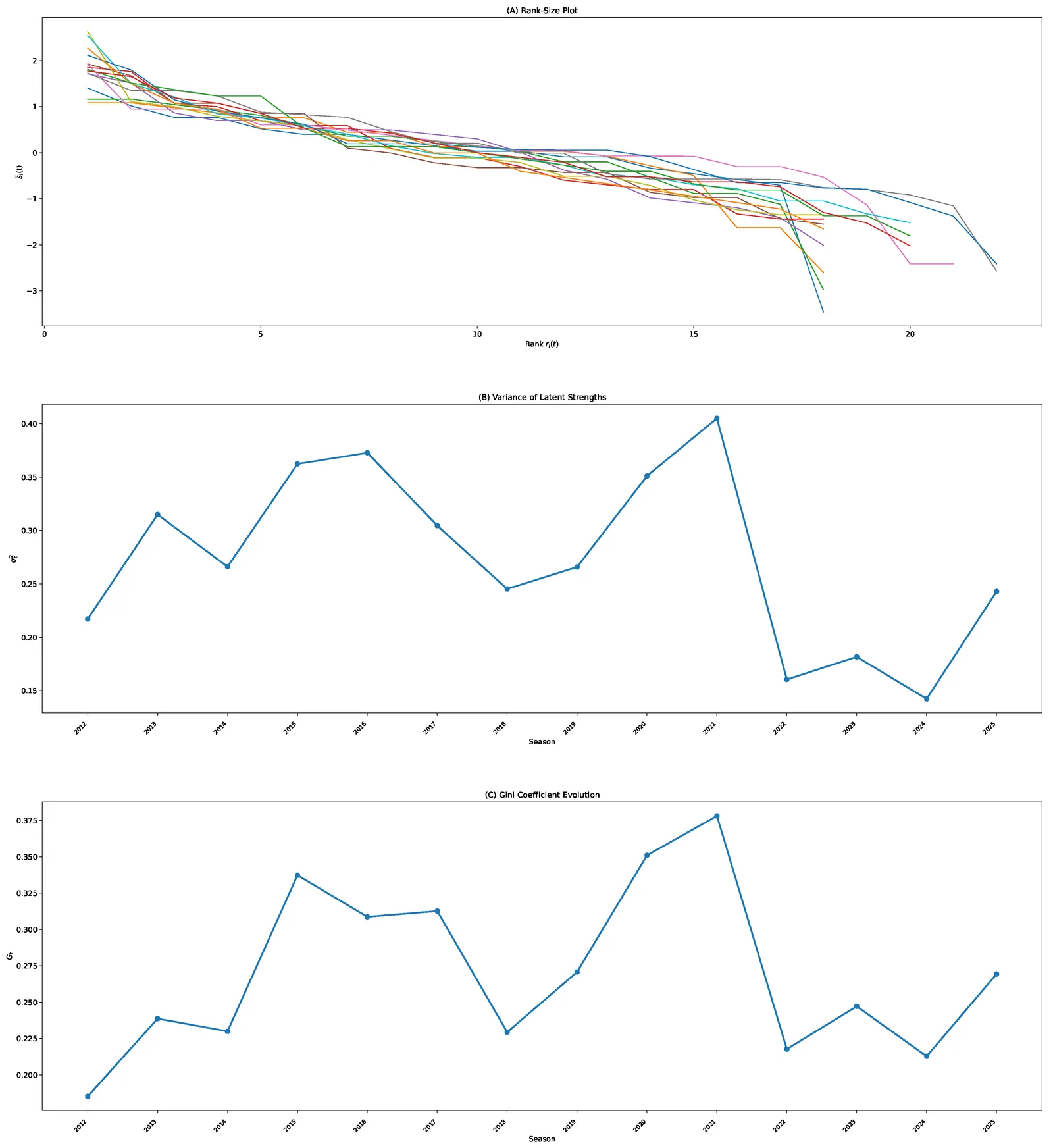
Hierarchical organization of {Japan_JLeague}: (**A**) rank-size visualizations of standardized latent strengths; (**B**) variance of non-standardized latent strengths; and (**C**) Gini coefficient of non-standardized latent strengths.

Figure 5 and Figure 6 show the results obtained for {England_Premier} and {Japan_JLeague}, respectively. Rank-size visualizations employ season-wise standardized latent strengths ${\tilde{s}}_{i}\left(t\right)$ in order to compare the geometric structure of competitive hierarchies across seasons. In contrast, variance and Gini observables are computed from the non-standardized latent strengths ${\hat{s}}_{i}\left(t\right)$, since these quantities are intended to measure the intrinsic magnitude of competitive dispersion and concentration within each season.

For both {England_Premier} and {Japan_JLeague}, the rank-size curves decrease faster across seasons at the top and bottom, while they are approximately linear in between. This indicates leagues with a small dominant elite teams, a more homogeneous middle tier, and high volatility between weaker teams, reflecting a core–periphery competitive structure. This effect is more pronounced in {England_Premier} than in {Japan_JLeague}, suggesting that hierarchy is weaker in the latter. For {England_Premier}, the variance of latent strengths exhibits a mild long-term increasing tendency, reaching pronounced peaks around the 2007–2008 and 2018–2019 seasons. This behavior indicates a temporary amplification of competitive stratification, corresponding to periods in which the separation between elite and lower-ranked teams became more pronounced. The Gini coefficient suggests an increasing competitive inequality trend and intermittent restructuring of competitive dominance. In contrast, for {Japan_JLeague} we see that the variance reaches its largest value around 2021, indicating a temporary strengthening of competitive differentiation during that period. Moreover, we verify that the Gini coefficient confirms this behavior. These results show that while both leagues have a fairly stable rank-size structure, they differ in how competitive inequality changes over time. {Japan_JLeague} shows more pronounced fluctuations in its hierarchy, while {England_Premier} shows a slower long-term rise in competitive concentration.

#### 5.1.4. Entropic Organization

**Figure 7:**
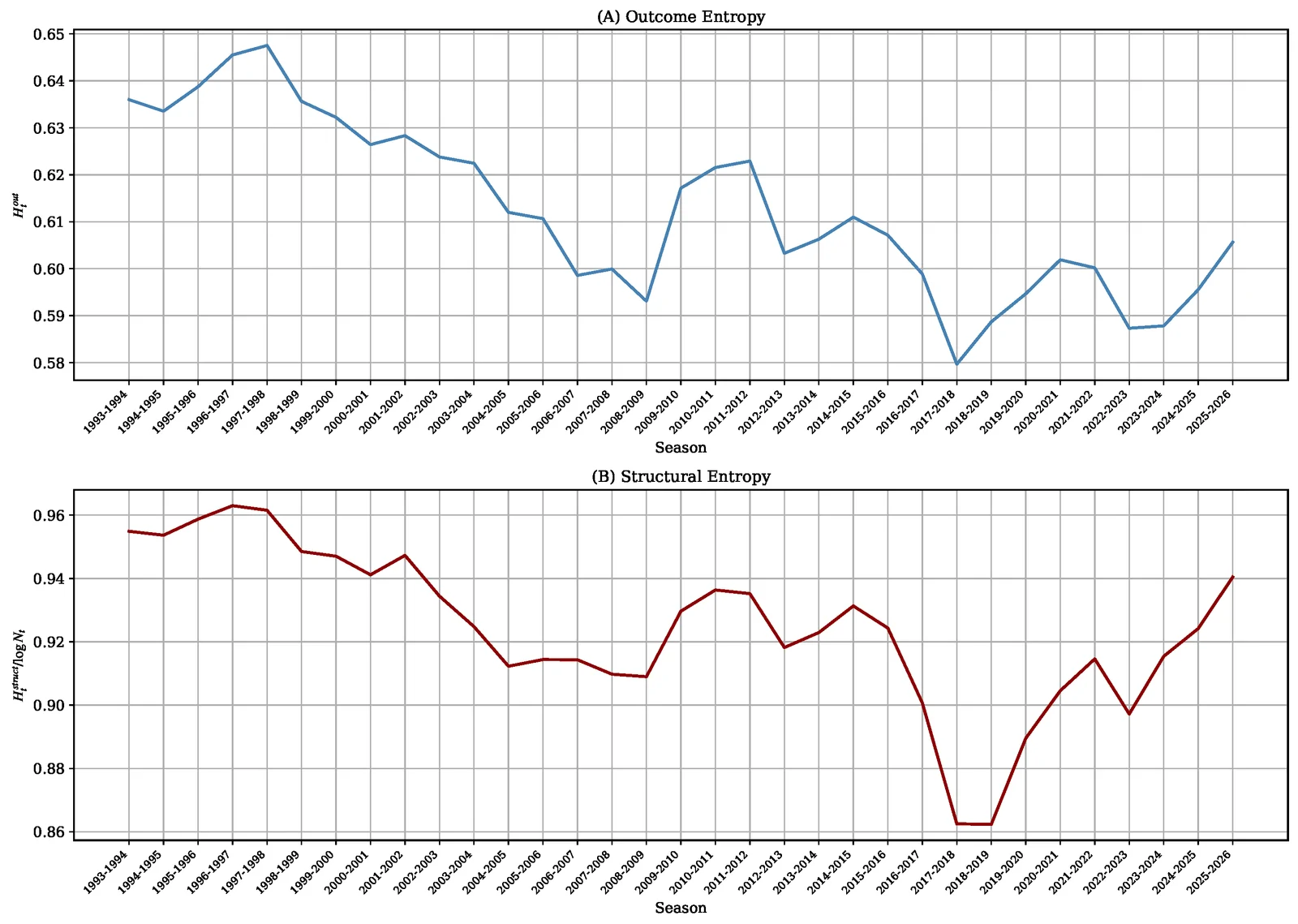
Entropic organization of {England_Premier}: (**A**) $H_{t}^{out}$ and (**B**) $\frac{H_{t}^{struct}}{logN_{t}}$.

**Figure 8:**
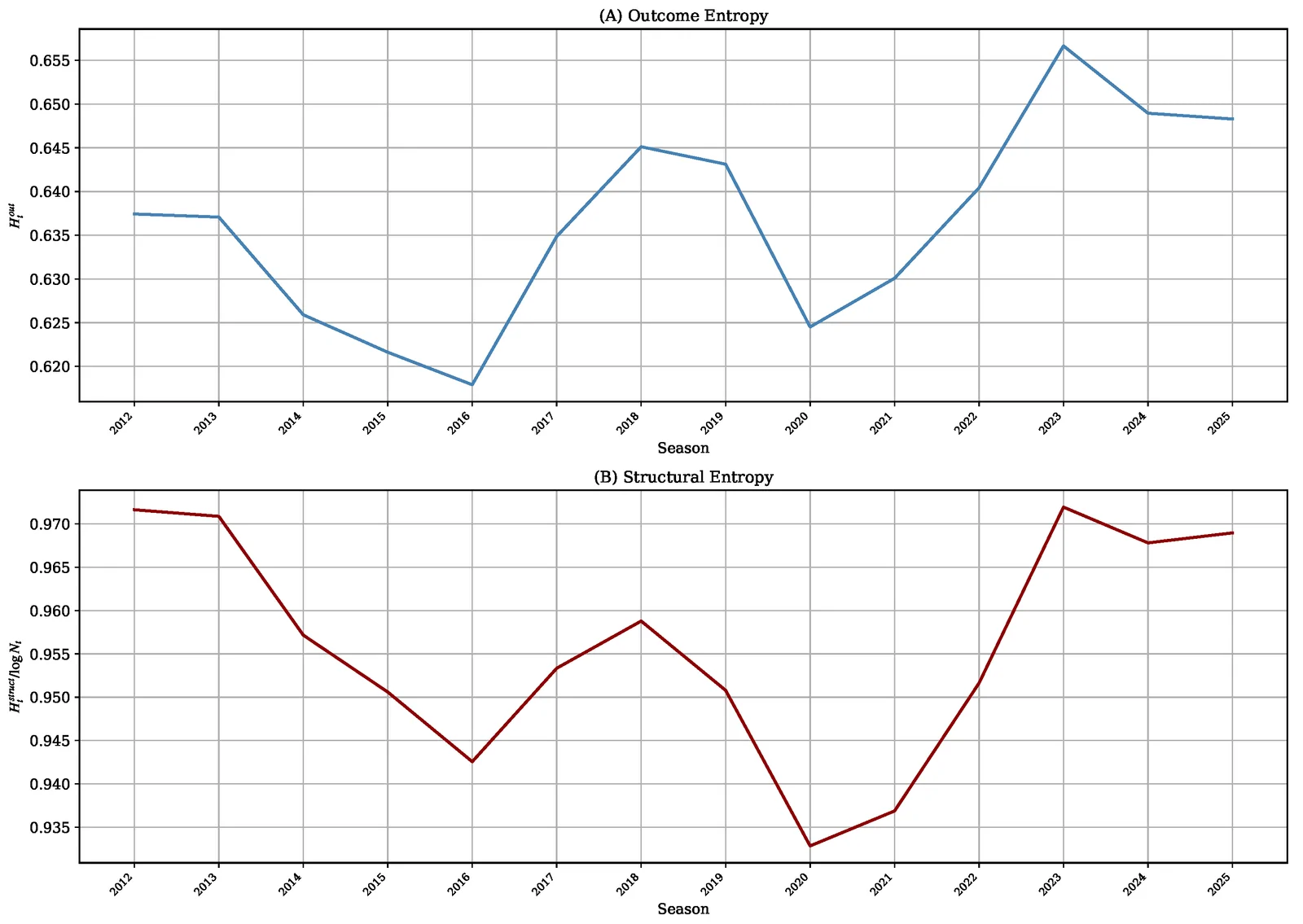
Entropic organization of Japan_JLeague: (**A**) $H_{t}^{out}$ and (**B**) $\frac{H_{t}^{struct}}{logN_{t}}$.

The entropy-based macroscopic observables include outcome entropy and structural entropy. Outcome entropy quantifies the uncertainty of match outcomes, meaning that it measures the degree of competitive unpredictability within the league. Structural entropy characterizes the configurational disorder of the latent strength distribution. Together, these indicators describe the balance between competitive order and disorder, enabling the identification of regimes characterized by high parity, strong hierarchy, or partially ordered states. Figure 7 and Figure 8 show $H_{t}^{out}$ and $H_{t}^{struct}$ normalized by the number of teams in each league season for {England_Premier} and {Japan_JLeague}, respectively.

The two macroscopic observables reveal quite different competitive dynamics between the leagues. For {England_Premier}, the outcome entropy exhibits a gradual decreasing trend through time, indicating that match outcomes became progressively more predictable and that competitive asymmetry increased over the period of analysis. The structural entropy also reveals a global decreasing trend, with two pronounced periods of reduction during 2002–2003, 2003–2004, and 2004–2005 and again during 2016–2017, 2017–2018, and 2018–2019, suggesting temporary increases in hierarchical concentration and strong dominance by a small subset of teams. By contrast, we verify that there is a slight increase trend in outcome entropy for {Japan_JLeague}. This indicates growing competitive unpredictability and a more balanced competitive environment over time. The structural entropy has a long-term stable trend but experiences temporary fluctuations, suggesting that the overall organization of competitive strength remained mostly uniform globally, though with some periods of change. This is evident in 2020 and 2021, when structural entropy changed more significantly, indicating a temporary reshaping of the competitive hierarchy and increased concentration effects during those seasons.

#### 5.1.5. Collective Competitive Order

We look at the overall collective order by using the concentration ratio of top teams and considering homefield advantage. The concentration ratio shows how much competitive power is focused within a small group of elite teams. The homefield advantage measures the overall imbalance in match outcomes and serves as an important external factor that affects how leagues operate. These large-scale measurements act as indicators of collective order, summarizing the main organizational features of the league.

**Figure 9:**
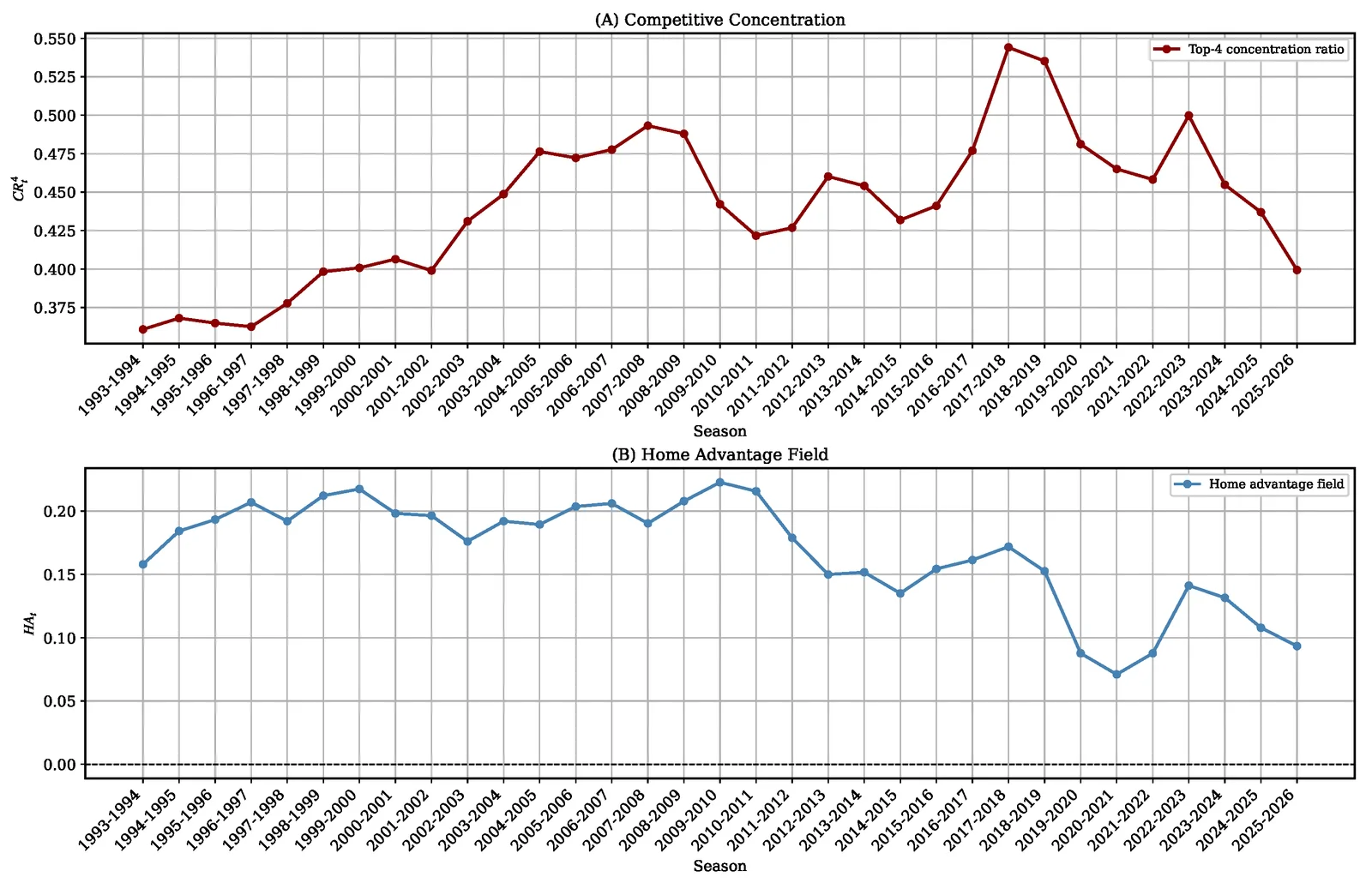
Collective competitive order of {England_Premier}: (**A**) $CR_{t}^{4}$ and (**B**) $HA_{t}$.

**Figure 10:**
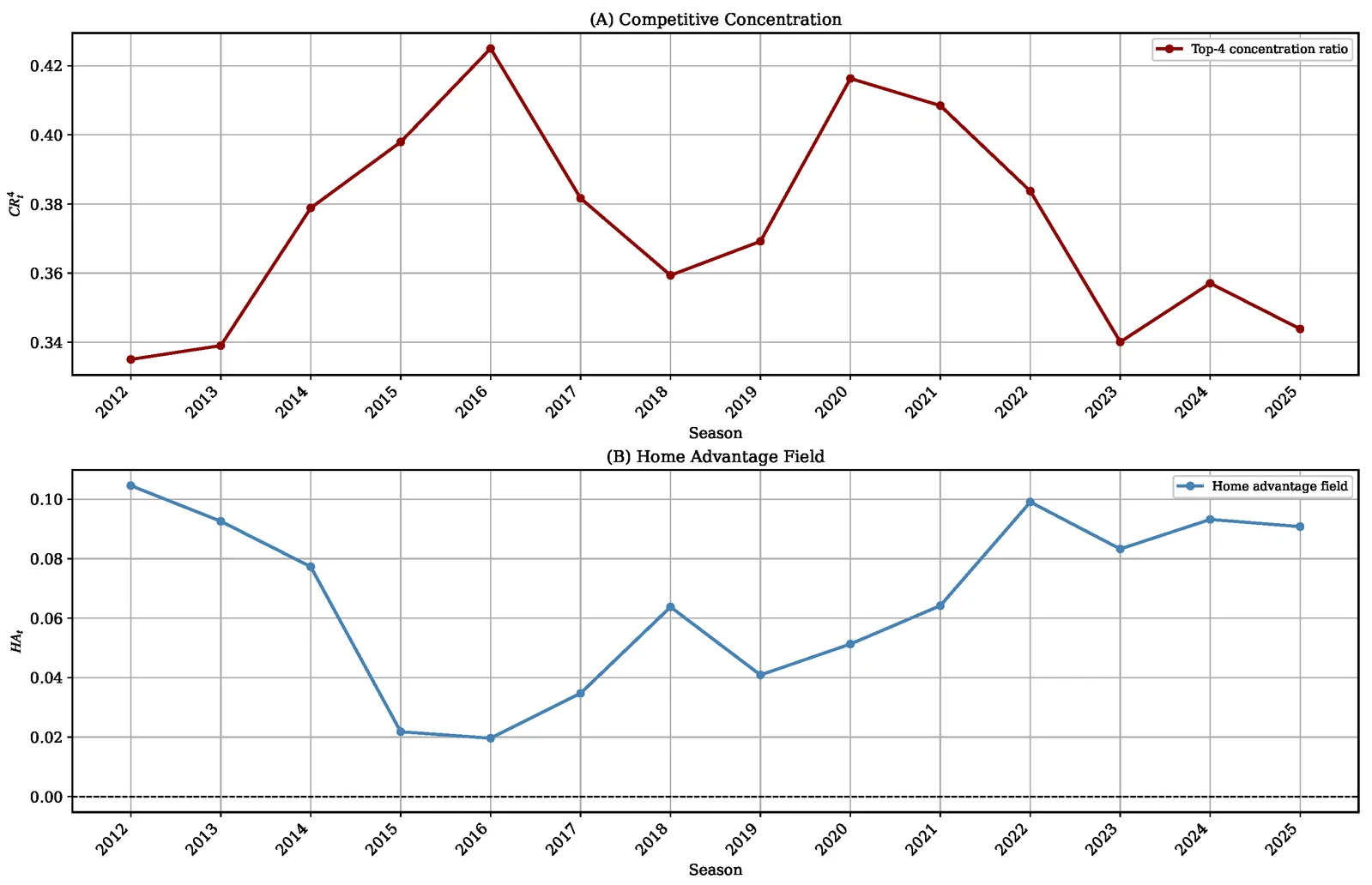
Collective competitive order of Japan_JLeague: (**A**) $CR_{t}^{4}$ and (**B**) $HA_{t}$.

Figure 9 and Figure 10 show the concentration ratio of the top four teams $CR_{t}^{4}$ and homefield advantage $HA_{t}$ for {England_Premier} and {Japan_JLeague}, respectively.

For {England_Premier} league, we see that the competitive concentration ratio changes in a nonlinear way. It rises until the 2008–2009 season, showing a growing concentration of power among the top teams. This rise is followed by a steady drop and stabilization until the 2016–2017 season, indicating a temporary shift in competitive strength throughout the league. There is a secondary increase from 2016–2017 to 2018–2019, after which concentration drops again. This pattern suggests a renewed spread of competitiveness. The homefield advantage remains fairly stable until around the 2010–2011 season, after which it shows a clear downward trend, pointing to a gradual weakening of home-field effects over time. A significant drop occurs during the 2020–2021 season, likely due to disruptions related to the COVID-19 pandemic and fewer spectators. For {Japan_JLeague}, the concentration ratio shows an oscillating trend. This indicates varying competitive strength among teams. Unlike {England_Premier}, the homefield advantage decreases until around 2016, after which we see a noticeable recovery. This suggests a partial return of homefield effects in the more recent seasons.

The hierarchical organization seen in the previous analysis shows how large-scale competitive structures arise from repeated microscopic interactions between teams. By breaking down latent-field dynamics, distributional geometry, hierarchical organization, entropy production, and collective order, the framework helps to identify ongoing structural patterns that are often overlooked in traditional league statistics.

The results show that both {England_Premier} and {Japan_JLeague} show persistent behavior analogous to symmetry breaking and long-term hierarchies, though their dynamics differ. In both leagues, the estimated strengths show differences between teams ${\hat{s}}_{i}\left(t\right)\neq {\hat{s}}_{j}\left(t\right)$, leading to stable competitive imbalances and ongoing hierarchy formation. The normalized strength trajectories, seasonal heatmaps, persistence relations, and rank-size structures clearly show the development of an ordered overall organization. The emergence of heterogeneous latent strengths can be interpreted heuristically as a symmetry-breaking process, in the sense that the initially indistinguishable competitive roles of the teams evolve into a hierarchical organization.

For {England_Premier}, the results show a stronger and more persistent hierarchical structure. The league has higher persistence of latent strengths, lower outcome uncertainty over time, temporary drops in structural uncertainty, and times of strong competitive concentration, especially in the mid-2000s. The decline in homefield advantage after 2010–2011 indicates a gradual reduction in the influence of homefield advantage on match outcomes. Despite periods of competitive redistribution, the league keeps a relatively stable hierarchical structure across seasons.

For {Japan_JLeague}, we see greater competitive mobility and weaker long-term persistence. Additionally, outcome entropy tends to increase, showing more competitive unpredictability, while structural entropy stays relatively stable except during 2020 and 2021. On the other hand, the rising concentration ratio and increasing Gini coefficient point to a gradual consolidation of competitive advantage among fewer teams. {Japan_JLeague} league combines higher local competitive variability with slowly rising overall stratification.

In both leagues, the computed distributions of normalized latent strengths remain quite stable across seasons. This indicates that there is persistence in the underlying organizational structure. Nevertheless, temporary intensification of competitive asymmetries and partial restructuring of league hierarchy seem to occur, as pointed out by the observed deviations associated with heavier tails, skewness, and increased variance emerging during specific periods.

Altogether, the obtained results are consistent with the interpretation that soccer leagues are a class of open evolving competitive systems viewed through a coarse-grained non-equilibrium perspective. Indeed, decreasing entropy, increasing variance, rising concentration measures, and stabilization of rank structures are consistent with the emergence of increasingly ordered macroscopic competitive organization.

#### 5.1.6. State-Space Trajectories

To describe how league organization changes over time, we look at state-space trajectories made from big-picture observables based on hidden competitive strengths. By showing how entropy, competitive concentration, variance, and homefield advantage change over time within simplified collective state-spaces, these state-space trajectories provide a visual representation of how leagues move between states of competitive balance, hierarchical strengthening, and structural changes.

The vector $X_{t}=\left(\sigma_{t}^{2},G_{t},H_{t}^{out},H_{t}^{struct},CR_{t}^{k},HA_{t}\right)\in R^{6}$ encodes the global structural properties of the league. Therefore, the temporal evolution of a league is described by a discrete trajectory $X_{t}\subset R^{6}$.

**Figure 11:**
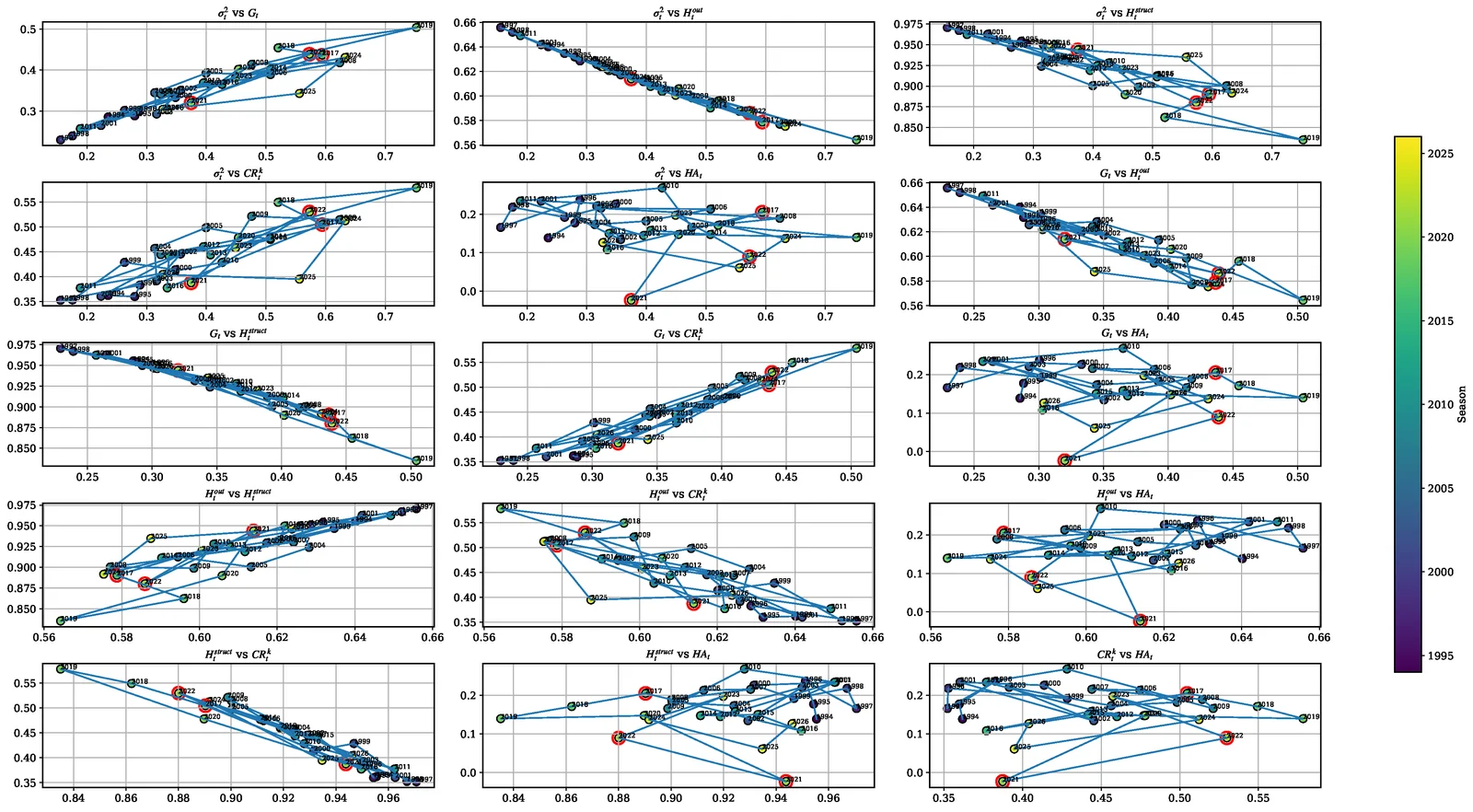
Evolution of {England_Premier}$X_{t}$, represented as 2D projections of all pairs of macroscopic variables.

**Figure 12:**
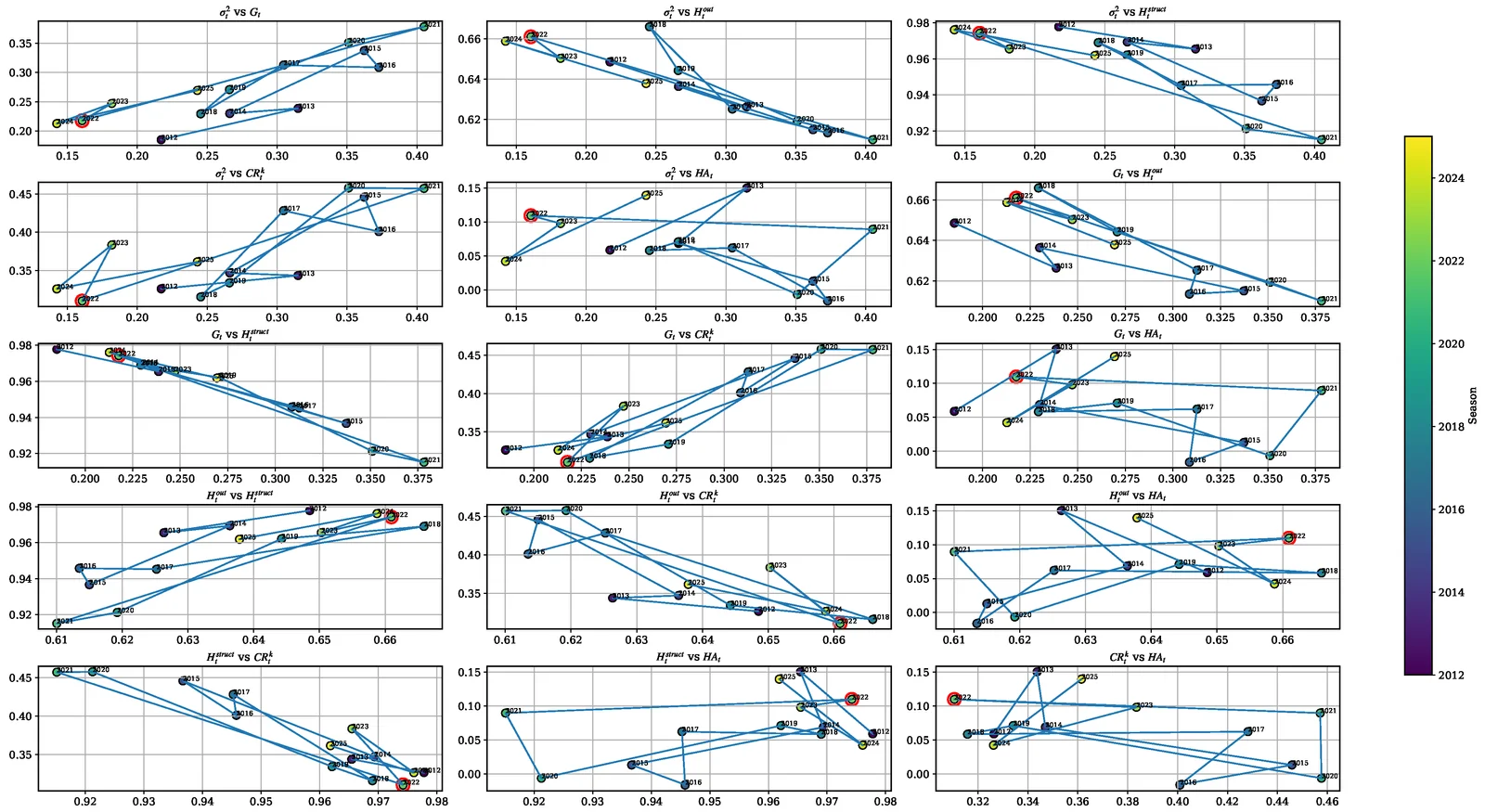
Evolution of {Japan_JLeague}$X_{t}$, represented as 2D projections of all pairs of macroscopic variables.

For visualization purposes, we consider all pairwise 2D projections of the full state-space, $\Pi_{t,pq}=\left(X_{t,p},X_{t,q}\right),\;p\neq q,\;p,q\in \left\{1,\ldots ,6\right\}$, as shown in Figure 11 and Figure 12 for {England_Premier} and {Japan_JLeague}, respectively. These projections provide interpretable slices of the underlying high-dimensional dynamics, but do not define independent dynamical systems; rather, they represent marginal views of a single trajectory evolving in $R^{6}$.

We define a structural transition as an anomalously large displacement in the full state-space $X_{t}$. The state variables possess different numerical scales and intrinsic variances. To ensure a balanced geometric contribution from all coordinates, each component is standardized across seasons using *z*-score normalization, yielding the normalized state vector:

(26) $${\tilde{X}}_{t}=\left({\tilde{x}}_{1,t},\ldots ,{\tilde{x}}_{6,t}\right).$$

We then compute the inter-seasonal increment:

(27) $$\Delta_{t}={\left\{\Delta \left(t\right)\right\}}_{t=1}^{T-1}={∥{\tilde{X}}_{t+1}-{\tilde{X}}_{t}∥}_{2}$$

where ${∥\;\cdot \;∥}_{2}$ denotes the Euclidean norm in $R^{6}$. A structural transition is identified whenever

(28) $$\Delta \left(t\right)>{\bar{\Delta }}_{t}+\alpha \;\cdot \;\sigma \left(\Delta_{t}\right),$$

with α=1.5.

For reliability, transitions are identified in 6D rather than in 2D projections. This avoids apparent displacements and projection artifacts that come up in 2D. As a result, structural transitions are computed in the standardized 6D state-space; 2D phase portraits are used only for visualization. For {England_Premier}, the results show transitions in the 2017–2018, 2021–2022, and 2022–2023 seasons (shown as 2017, 2021, and 2022 in the figures to minimize text overlapping). For {Japan_JLeague}, we verify that a transition occurred in 2022. These transitions are associated with changes in the macro-state ${\tilde{X}}_{t}$, corresponding to shifts in the structural properties of the macroscopic observables.

#### 5.1.7. Bootstrap Uncertainty of Macroscopic Observables

Because latent strengths are inferred from a finite number of matches, both the estimated strengths and the derived macroscopic observables are subject to sampling variability. To quantify this uncertainty, a bootstrap-based robustness analysis was performed by propagating match-level uncertainty through the complete inference pipeline.

**Figure 13:**
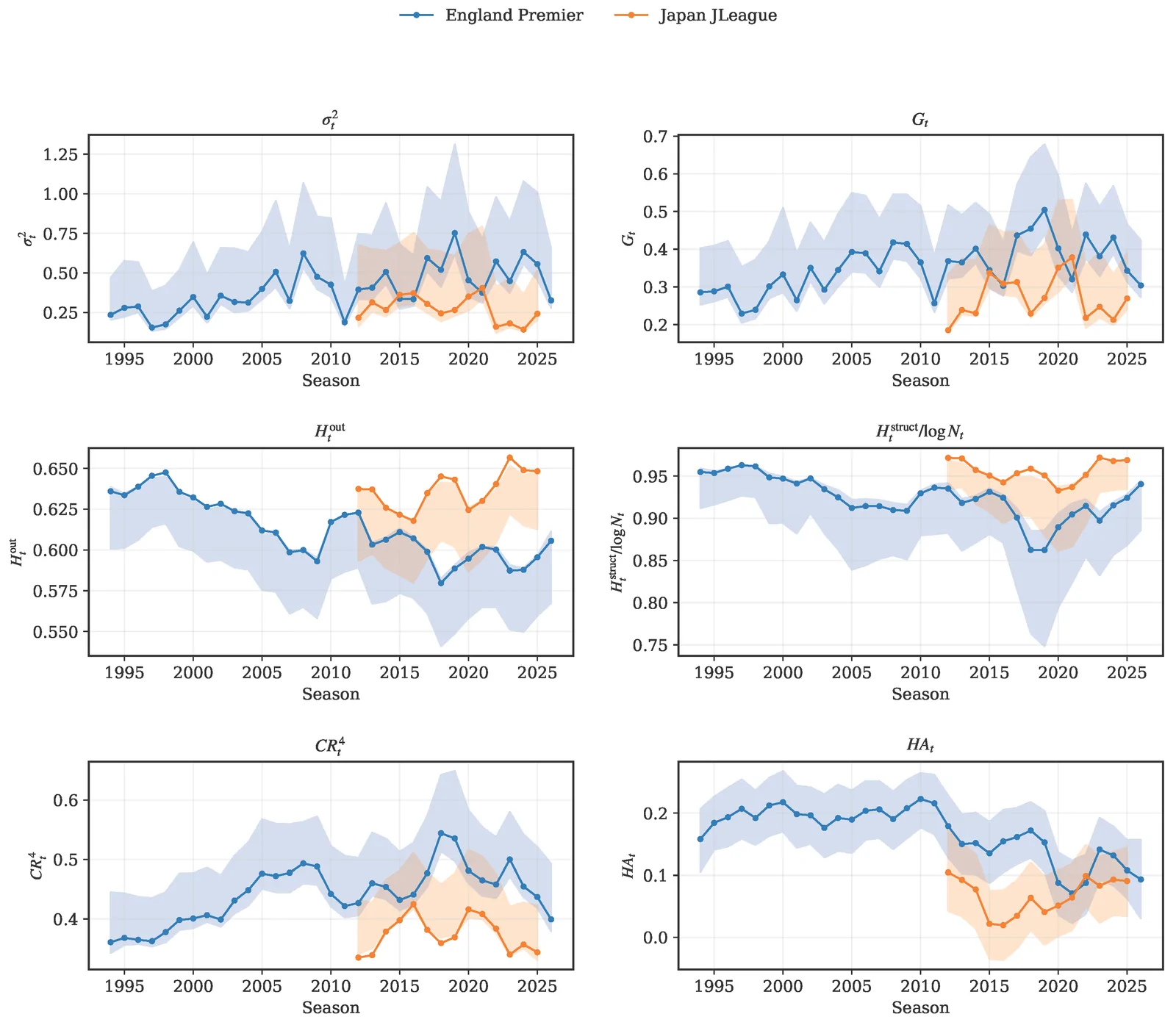
Bootstrap 95% CIs for the six seasonal macroscopic observables in the representative leagues {England_Premier} and {Japan_JLeague}. Solid lines indicate the original seasonal estimates; the shaded areas are the 95th percentile bootstrap CIs.

For each league season, bootstrap resampling was carried out by sampling the matches with replacement while keeping the original number of matches. A number of 500-bootstrap replicates were produced, and the entire inference pipeline, encompassing the estimation of the latent team strengths and the recalculation of all six macroscopic observables, was performed for each of the bootstrap replicates. This involved re-estimating the Bradley–Terry model (with an $L^{2}$ regularization parameter of λ=0.1), centering the latent team strengths, and recalculating the six seasonal observables. Bootstrap means, standard errors, and 95th percentile confidence intervals (CIs) were retained for every seasonal observable. Figure 13 illustrates the resulting CIs for the representative leagues {England_Premier} and {Japan_JLeague}, while Table 2 summarizes the median bootstrap standard errors and CI widths across all observables. The bootstrap CIs indicate that the principal temporal patterns of the seasonal observables are preserved under match-level resampling. Although the magnitude of sampling uncertainty varies across observables and league seasons, the overall temporal evolution remains stable.

### 5.2. Cross-League Comparison

Although soccer leagues vary greatly in geography, financial structure, competitive culture, league size, and history, they might still show similar overall patterns of behavior. To explore this idea, we depict each league as a path in a shared state-space made from scale-invariant measures that reflect different aspects of competitive organization. This approach enables us to compare systems that are structurally different in a systematic way.

#### 5.2.1. Normalized Macro-State Representation

For each league L and season *t*, we define a macroscopic state vector

(29) $$X_{L,t}=\left(\sigma_{L,t}^{2},G_{L,t},H_{L,t}^{\mathrm{out}},H_{L,t}^{\mathrm{struct}},CR_{L,t}^{k},HA_{L,t}\right)\in R^{6},$$

which captures different aspects of league organization. Each component is either dimensionless by design or adjusted based on match counts or system size. This setup allows for comparison across leagues that have different numbers of teams and fixtures.

To reduce short-term fluctuations and isolate persistent structural characteristics, we define the league-level macro state as the temporal average over available seasons:

(30) $$X_{L}^{\left(i\right)}=\frac{1}{T_{L}}\sum_{t=1}^{T_{L}}X_{L,t}^{\left(i\right)},\;i=1,\ldots ,6$$

where $T_{L}$ denotes the number of observed seasons in league L. This aggregation collapses time and yields a single representative point in macro state-space, summarizing the long-run organizational regime of each league.

Despite the $X_{L}$ components being dimensionless, the empirical distributions of the variables differ across leagues. For effective geometric comparison across systems, we perform cross-league standardization. For each observable i∈{1,…,6}, we compute

(31) $${\tilde{X}}_{L}^{\left(i\right)}=\frac{X_{L}^{\left(i\right)}-\mu_{i}}{\sigma_{i}},$$

where $\mu_{i}$ and $\sigma_{i}$ stands for the cross-league mean and standard deviation of the *i*-th macro state coordinate, respectively, and ${\tilde{X}}_{L}\in R^{6}$ is the normalized vector.

This procedure ensures that Euclidean distances in macro state-space reflect relative deviations from global structural patterns rather than absolute scale differences between leagues. Consequently, comparisons across leagues become geometrically meaningful and invariant to differences in measurement scale.

#### 5.2.2. Shared Macroscopic Organization Pipeline

Within the framework adopted in this paper, universality refers to shared macroscopic organization, meaning that leagues with different historical, geographical, and institutional characteristics occupy similar regions of the proposed macro state-space. We do not claim universality in the strict statistical-physics sense associated with critical phenomena, scaling laws, or invariant critical exponents. When leagues populate similar regions of ${\tilde{X}}_{L,t}\in R^{6}$, they follow similar trajectories or possess analogous geometric organization; in other words, despite differences in teams, countries, economic environments, and historical contexts, the leagues’ large-scale dynamics can be regarded as manifestations of common underlying mechanisms.

Despite match outcomes being uncertain and league compositions changing over time, large-scale stable patterns can still emerge. Thus, the existence of common geometric patterns, entropy, and structures across leagues will suggest evidence of common macroscopic organization.

To investigate the above hypothesis, a sequence of complementary analyses is performed:

1. Construction of the normalized macro state-space ${\tilde{X}}_{L,t}\in R^{6}$ and analysis of pairwise projections.
2. Principal component analysis (PCA) and estimation of effective dimensionality.
3. Nonlinear dimensionality reduction using uniform manifold approximation and projection (UMAP).
4. Distance-based comparison and hierarchical clustering of the league-level macro state ${\tilde{X}}_{L}\in R^{6}$.
5. Regime detection in the league-level macro state.
6. Detection of macroscopic organization based on geometric and dynamical similarity.

The ordering goes from representation to inference: first, we establish the geometry of the seasonal macro-state space; then, we look into its dimensional structure and how it is organized; next, we compare leagues using clustering and distance-based methods on league-level macro state; finally, we identify dynamical regimes and interpret the resulting structures in terms of common macroscopic organization classes.

**Figure 14:**
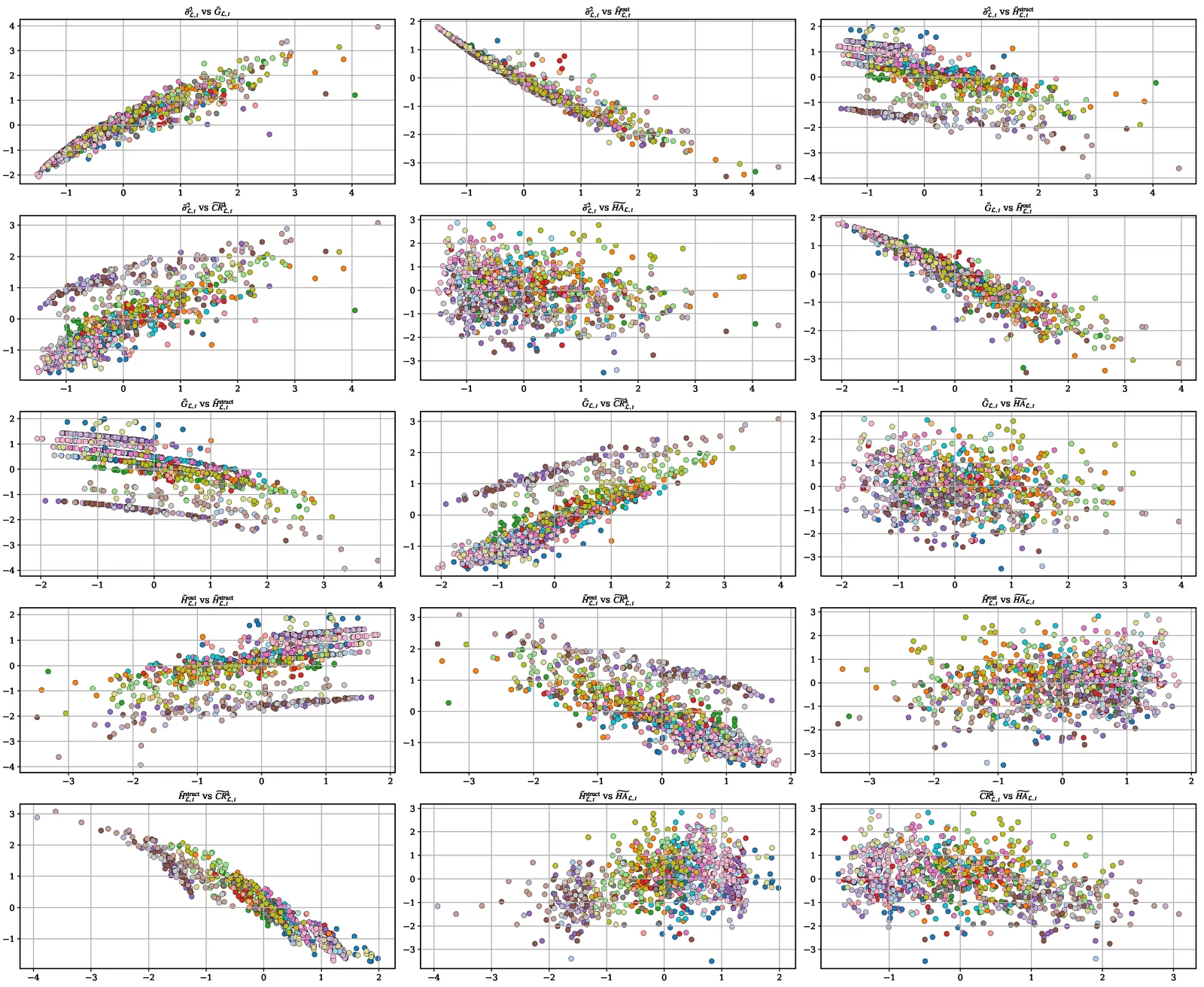
The 2D projections of ${\tilde{X}}_{L,t}$ of all leagues.

The pairwise projections of ${\tilde{X}}_{L,t}$ of all leagues, shown in Figure 14, indicate that the 6D state-space is not evenly filled. Instead, observations are concentrated in specific areas with strong correlations among several macro state variables. Projections that include $\left\{{\tilde{\sigma }}_{L,t}^{2},{\tilde{G}}_{L,t}\right\}$, $\left\{{\tilde{G}}_{L,t},{\tilde{CR}}_{L,t}^{k}\right\}$, and $\left\{{\tilde{H}}_{L,t}^{\mathrm{out}},{\tilde{H}}_{L,t}^{\mathrm{struct}}\right\}$ point to the presence of distinct regions (clusters of macro states) within the state-space. These structured patterns show that league evolution is limited by underlying organizational mechanisms. The effective dynamics only occupy a portion of the available 6D macro state-space.

**Figure 15:**
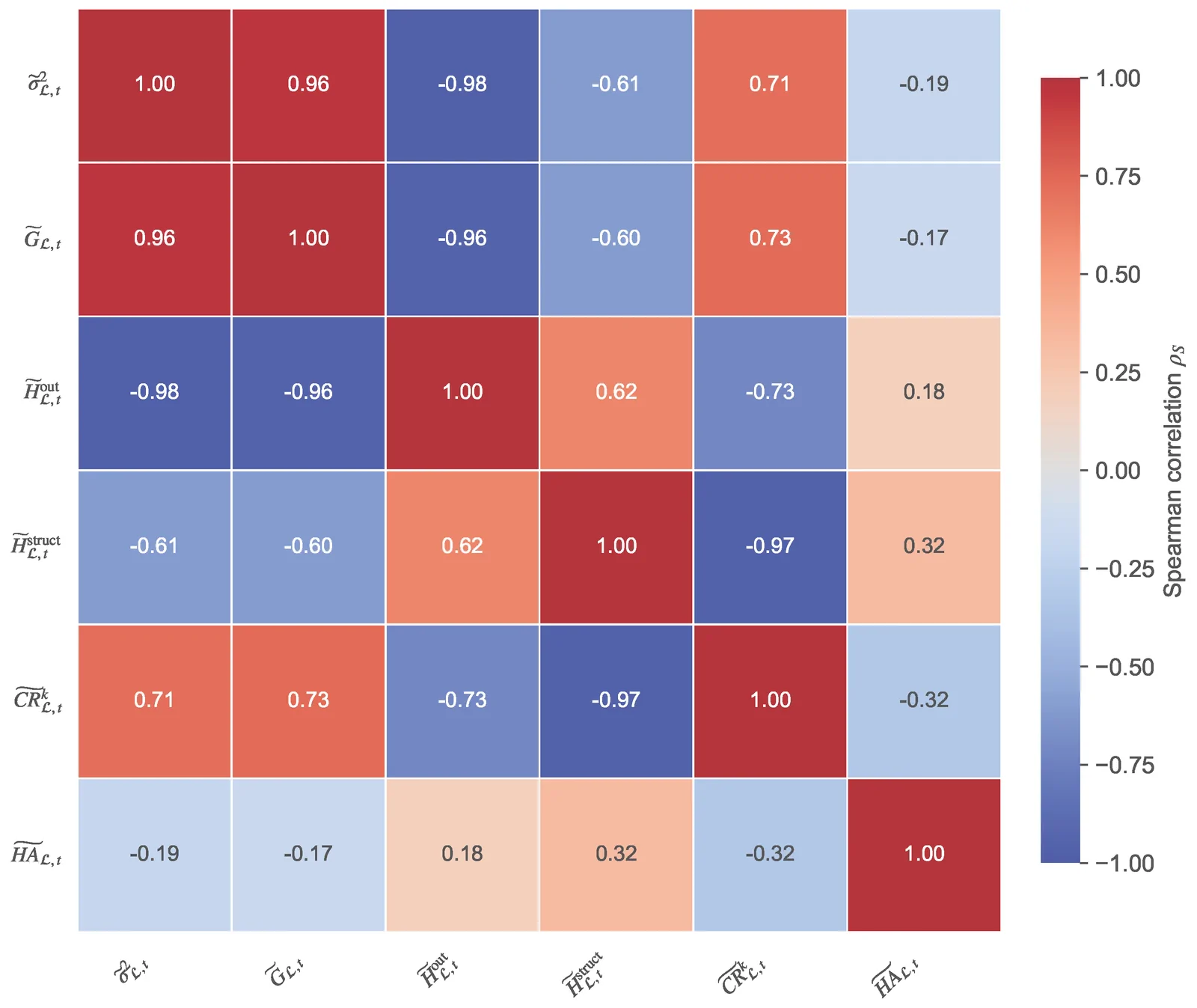
Spearman correlation matrix of the six standardized seasonal macro state observables computed over all league season states ${\tilde{X}}_{L,t}$.

The normalized macro state-space ${\tilde{X}}_{L,t}\in R^{6}$ describes related but distinct aspects of league season organization. To quantify the empirical dependence, we compute the Spearman correlation matrix over all league season macro states. Figure 15 depicts the correlation matrix.

We can verify the existence of substantial statistical dependence among several observables. In particular, there are strong correlations between variance and outcome entropy ($\rho_{S}=-0.982$), variance and Gini coefficient ($\rho_{S}=0.964$), and structural entropy and concentration ratio ($\rho_{S}=-0.968$). These $\rho_{S}$ values are expected, since these observables quantify closely related aspects of the inferred latent-strength distribution. Nevertheless, all six variables are retained in order to preserve the conceptual integrity and interpretability of the macro state representation. Each observable characterizes a different aspect of league organization, including strength dispersion, competitive inequality, outcome uncertainty, structural organization, concentration of competitive strength, and homefield advantage. Eliminating variables solely because they are correlated could remove meaningful distinctions between descriptors that exhibit similar statistical behavior while representing different organizational properties. Accordingly, we regard the six-dimensional macro state as an intentionally overcomplete yet interpretable representation in which statistical redundancy reflects overlapping but conceptually distinct facets of competitive organization.

**Figure 16:**
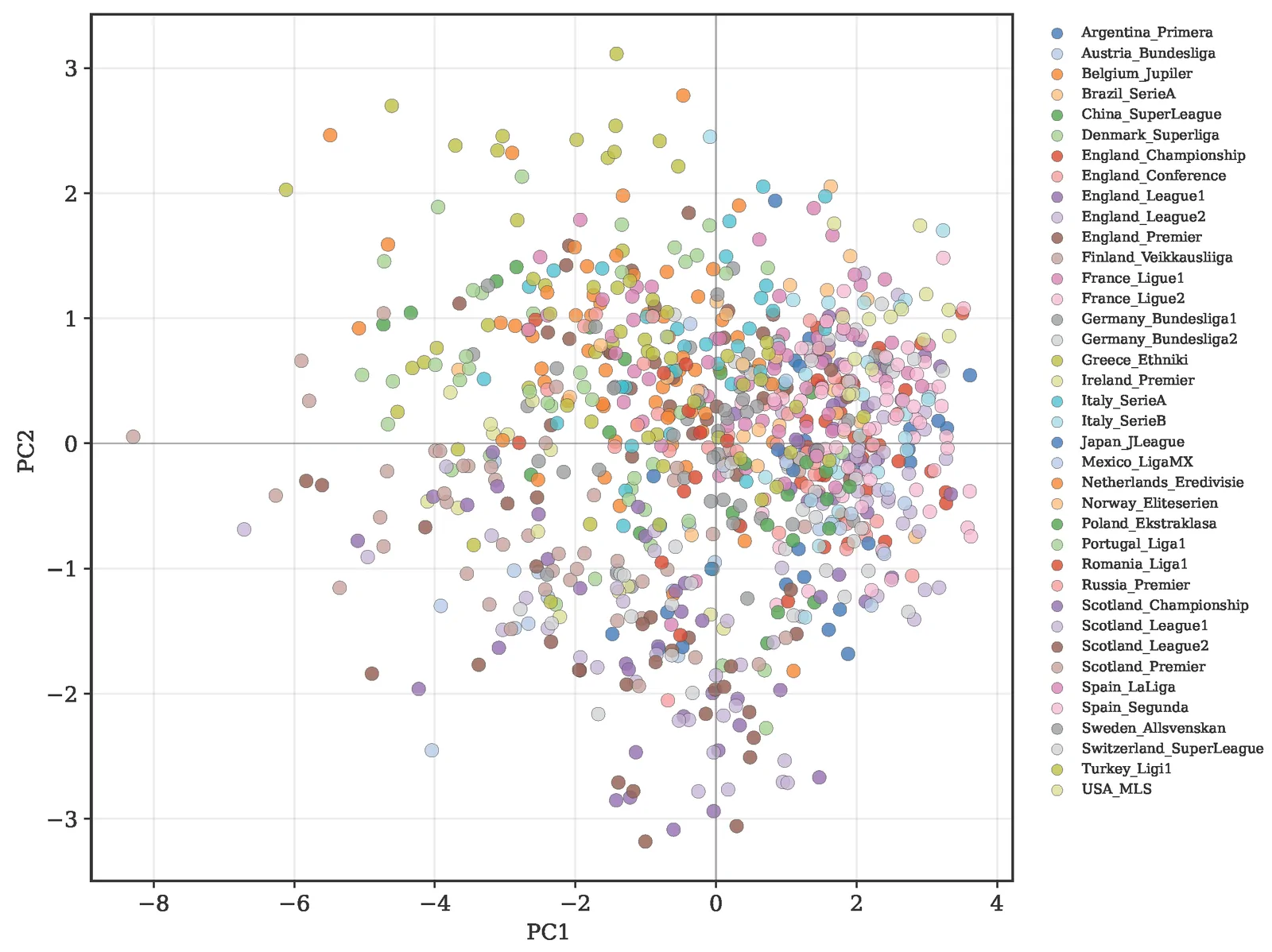
PCA projection of the seasonal macro state-space of all leagues, ${\tilde{X}}_{L,t}$.

**Figure 17:**
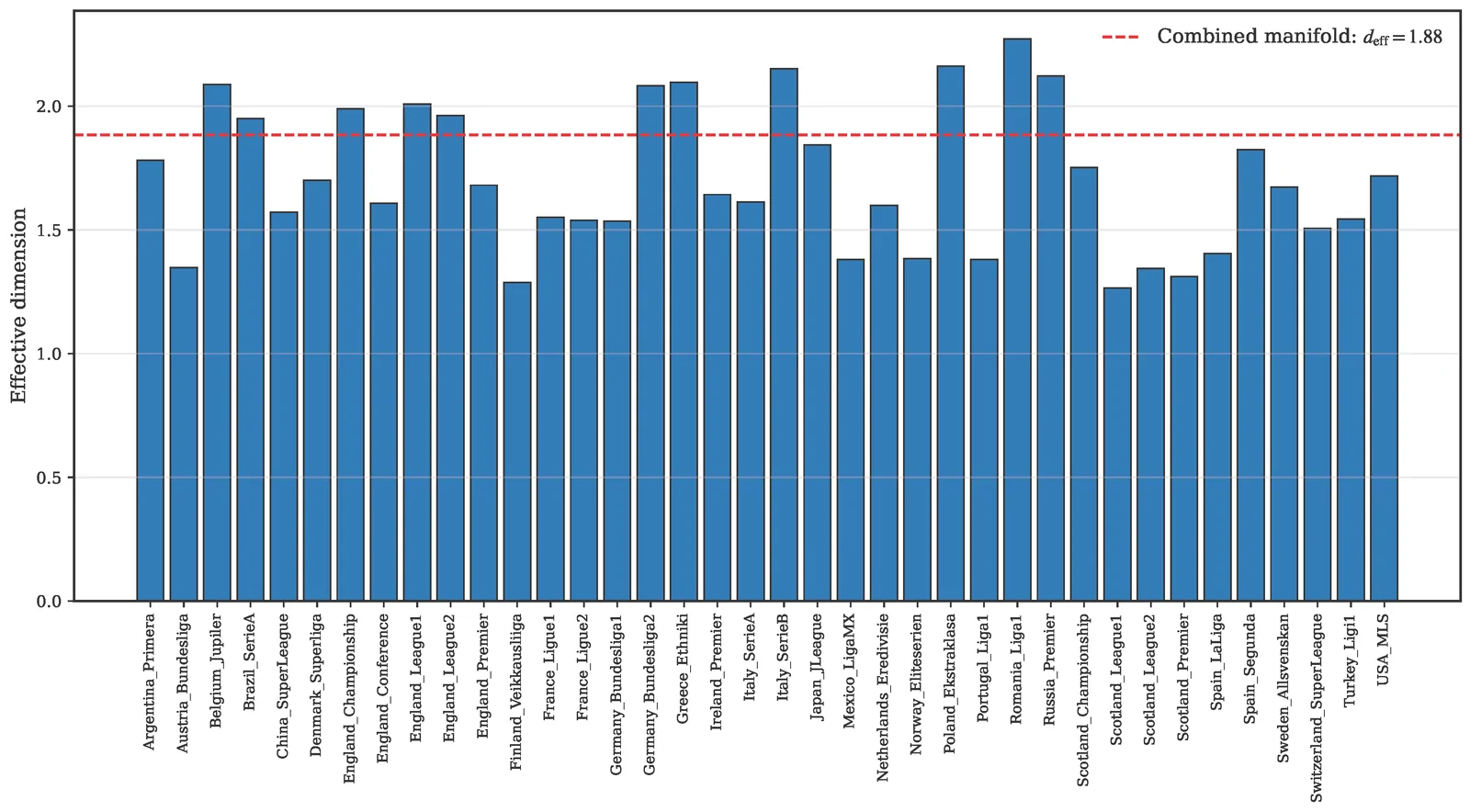
Effective dimensionality of ${\tilde{X}}_{L,t}$.

To further investigate the effective dimensionality of the macro-state, we performed a PCA. Figure 16 illustrates the seasonal macro-state representation. As shown in Figure 17, the seasonal macro states of individual leagues are dominated by one or two principal modes of variation, indicating that their variability can be well approximated by a low-dimensional representation. When the seasonal macro states of all leagues are analyzed jointly, they exhibit low effective dimensionality ($d_{\mathrm{eff}}=1.88$), suggesting that most of the variability is concentrated in a small number of collective modes. Approximately 90% of the total variance is captured by the first two principal components, indicating that the six-dimensional macro state representation can be summarized by a small number of dominant modes. This reflects the variety of the trajectories and macro state arrangements among the competitions while at the same time indicating that they lie in a common low-dimensional area of the original 6D macro state-space.

**Figure 18:**
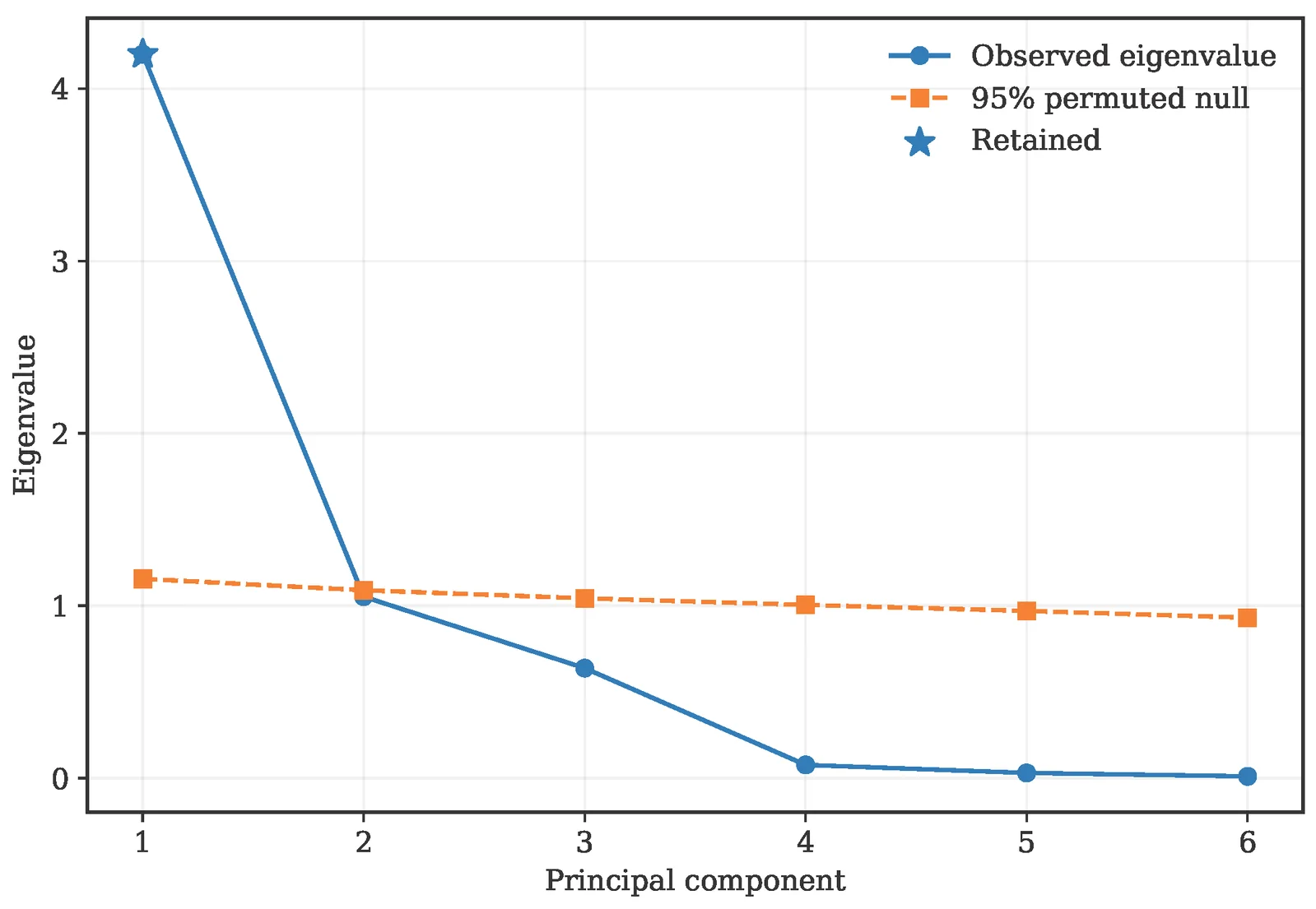
Horn’s parallel analysis of the seasonal macrostate PCA. Principal components are retained when the observed eigenvalue exceeds the 95th percentile of the permutation null distribution.

**Figure 19:**
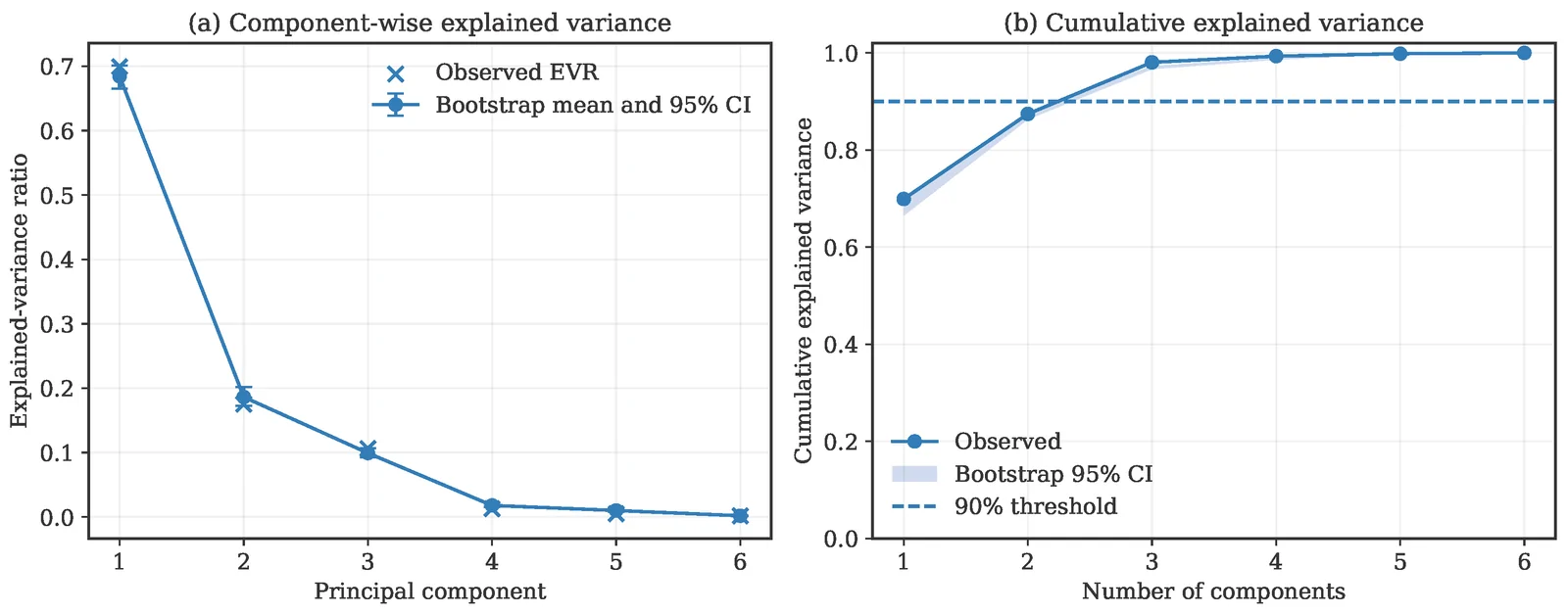
Bootstrap stability of the PCA explained variance ratios (EVRs). Error bars represent 95th percentile CIs obtained from 500 bootstrap replicates.

The robustness of the PCA solution was evaluated using the 500 bootstrap macrostate datasets. For each bootstrap replicate, the normalized macrostate matrix was analyzed using the same PCA procedure as in the original analysis. Explained variance ratios and component loadings were retained for every replicate, allowing bootstrap CIs to be estimated. Horn’s parallel analysis was additionally performed using 1000 independently permuted datasets, and principal components were retained when their observed eigenvalues exceeded the 95th percentile of the permutation null distribution. Figure 18 presents the parallel analysis, Figure 19 summarizes the bootstrap stability of the explained variance, and Table 3 reports the numerical validation statistics.

Horn’s parallel analysis retained only the first principal component, whereas the effective $d_{\mathrm{eff}}=1.88$ indicates that the overall variance is distributed across approximately two dominant modes. Together, these results support a low-dimensional representation of the seasonal macro state while highlighting that only the leading principal component is statistically distinguishable from the permutation null model.

**Figure 20:**
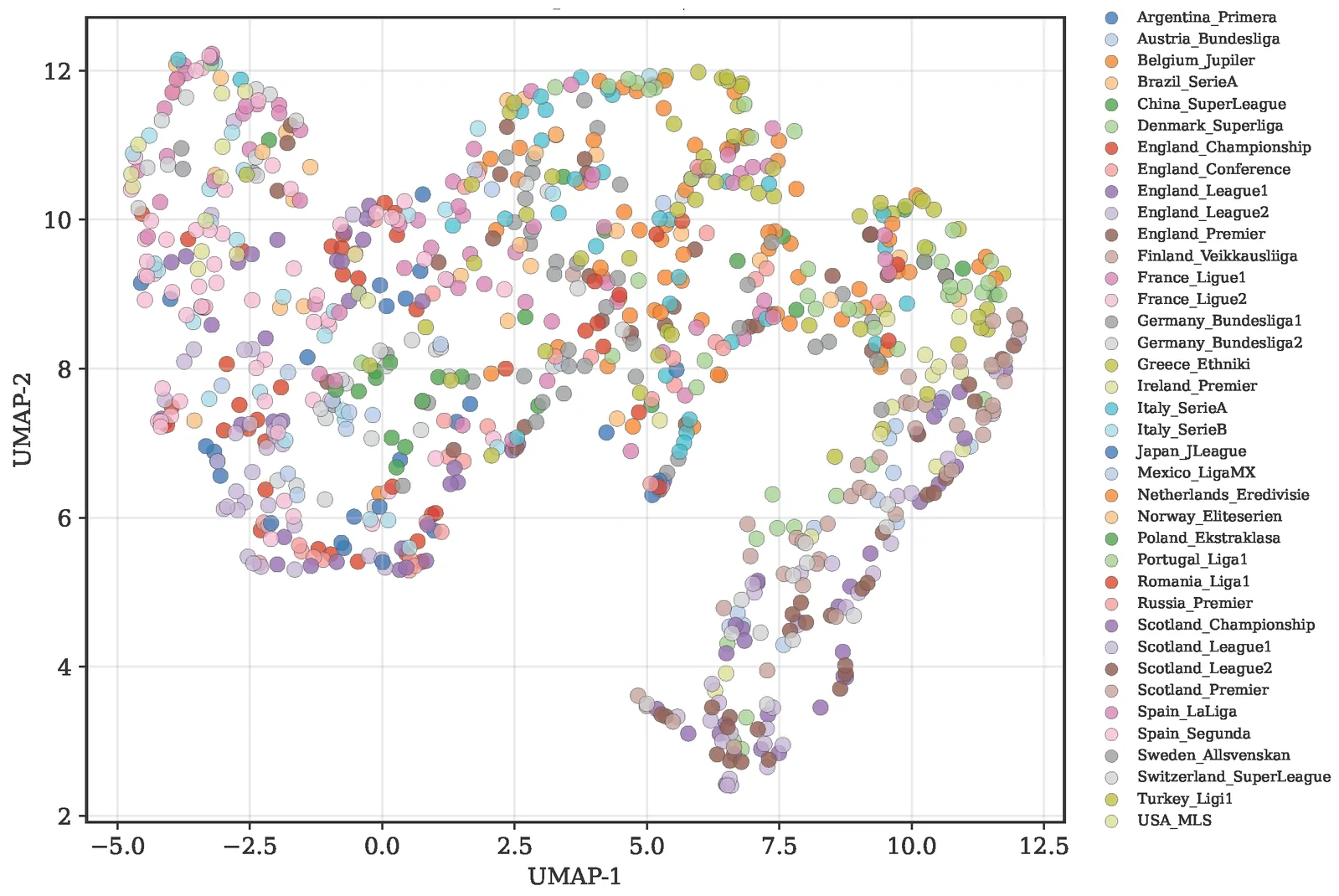
Two-dimensional UMAP projection of the seasonal macro state-space ${\tilde{X}}_{L,t}$.

The nonlinear UMAP embedding illustrated in Figure 20 serves as a complementary way of visualizing the low-dimensional macro state structure exposed by the PCA. It shows one main cluster, a smaller cluster, and a group of systems linking the two, which indicates that the league season macro states are fairly organized. The fact that this organization is in agreement with that seen in the PCA representation means that the principal relationships between the macrostates are retained in both the linear and nonlinear projections. Because the robustness of the low-dimensional structure was established through the bootstrap validation and parallel analysis of the PCA solution, the UMAP embedding is interpreted as an exploratory visualization that facilitates the interpretation of the macro state landscape rather than as an independent source of statistical evidence.

**Figure 21:**
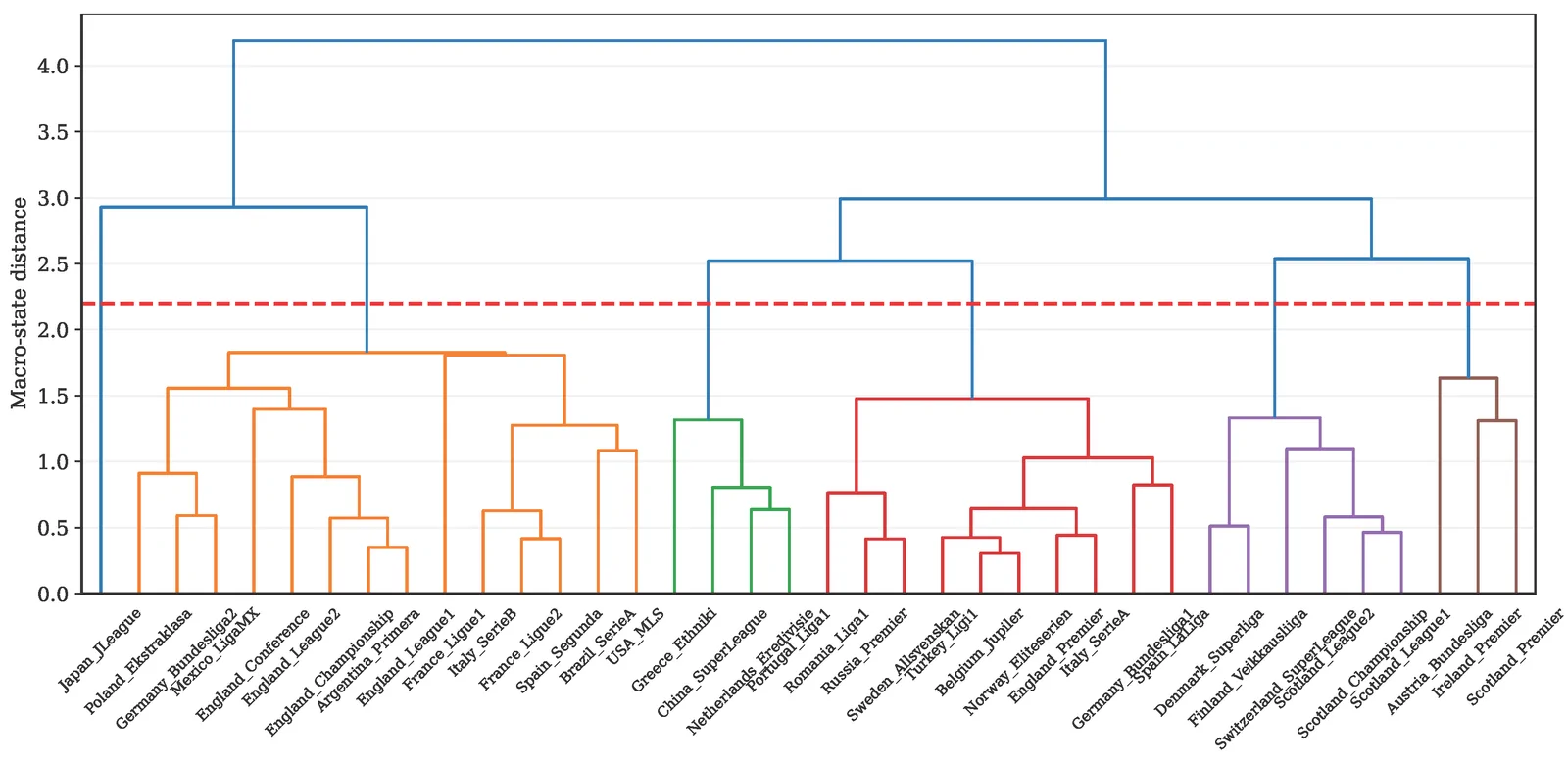
Dendrogram of ${\tilde{X}}_{L}$ distances.

To further investigate similarities between leagues, seasonal macro states are aggregated into league-level macro states and pairwise distances are computed in the standardized league-level macro state-space ${\tilde{X}}_{L}$. The hierarchical clustering dendrogram shown in Figure 21 reveals that the leagues do not show random distribution throughout ${\tilde{X}}_{L}$. Instead, we verify the presence of four major clusters and possible outliers.

**Figure 22:**
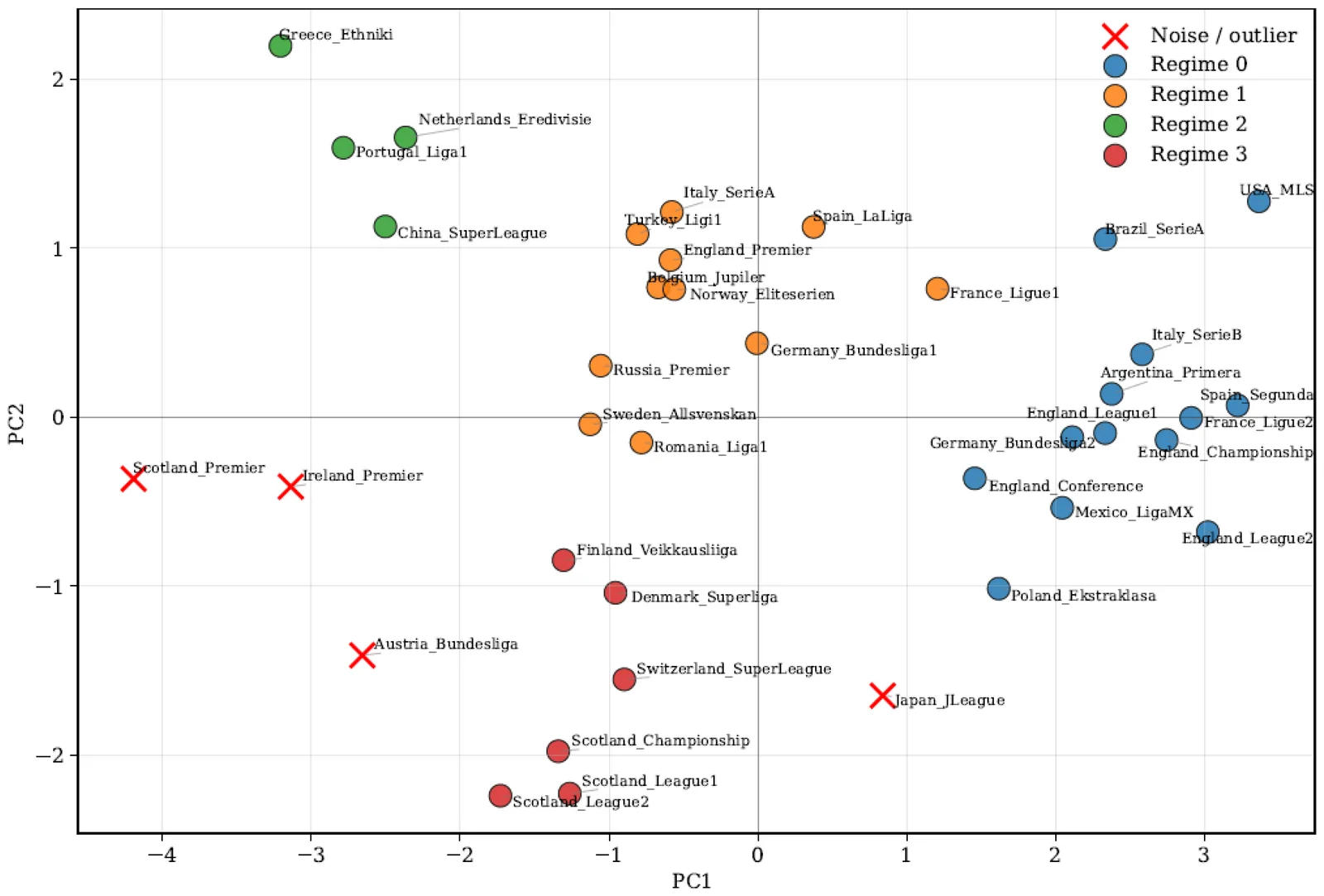
DBSCAN clustering of leagues in the full 6D macro state-space, visualized in the PCA representation.

A complementary perspective is provided by using a density-based clustering procedure (DBSCAN) in the full 6D ${\tilde{X}}_{L}$ space. We notice the presence of four dominant groups in terms of structural characteristics, together with a few isolated leagues that behave as outliers. Figure 22 depicts the corresponding PCA 2D projection. Although the precise classification depends on the clustering methodology, both approaches consistently indicate that leagues organize into a small number of well-defined geometric groups. Hierarchical clustering uncovers nested similarities among leagues. DBSCAN in the full 6D space captures fine-grained patterns of variability in the league-level macro state representation.

**Figure 23:**
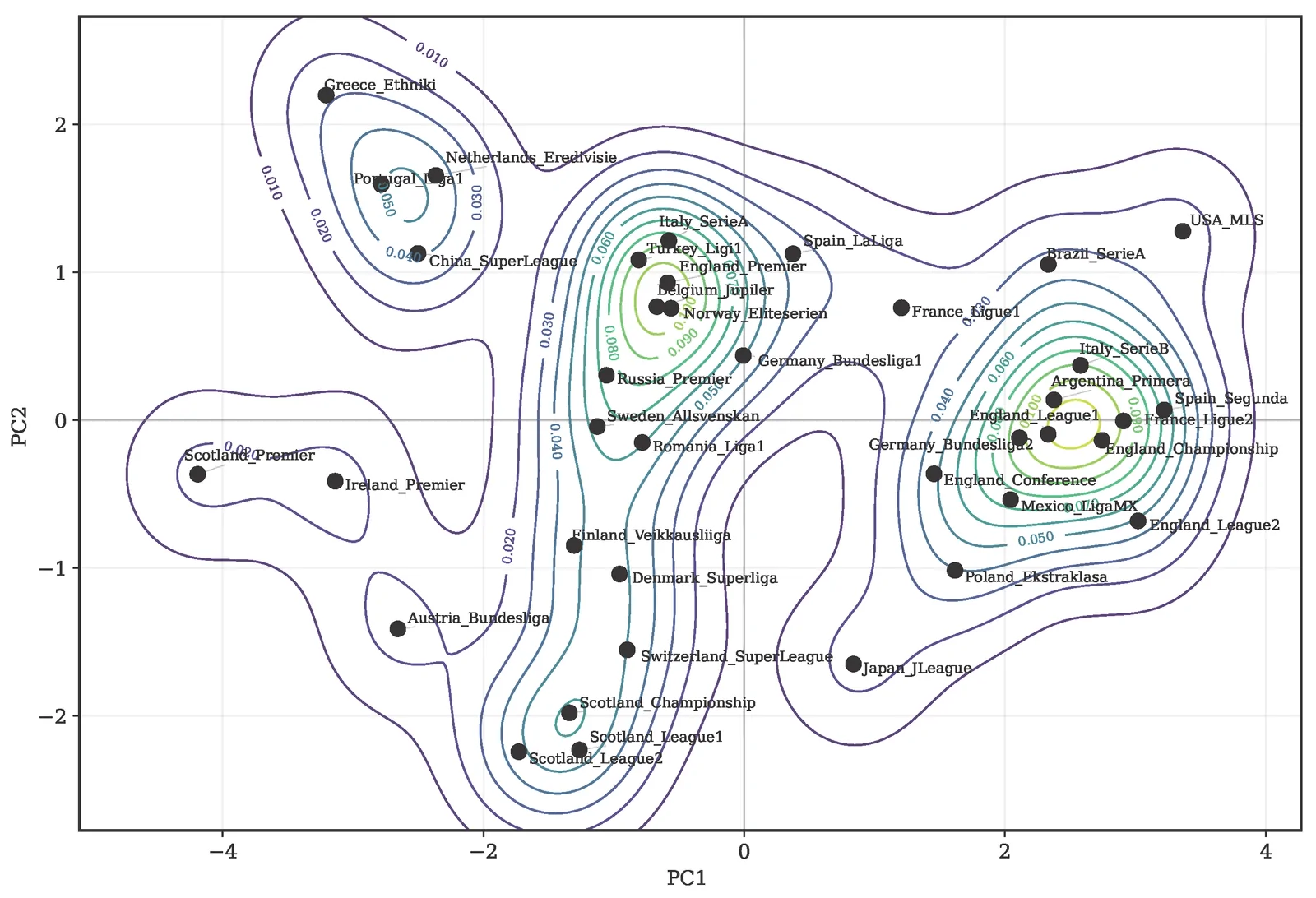
Kernel density representation of leagues in the coarse-grained PCA representation.

As an alternative interpretation, the league-level macro state is projected onto a 2D space using PCA to obtain a coarse-grained representation of the competitive landscape and a kernel density estimation (KDE) is applied in 2D to characterize the continuous phase-space distribution (density field) of leagues. This provides a notion of emergent large-scale organization as opposed to discrete macroscopic organization classes. The results in Figure 23 reveal a predominant density structure composed of two high-density regions, two secondary lower-density regions, transition areas, and possible outliers. Comparing Figure 22 and Figure 23, we verify that DBSCAN in full 6D macro state-space identifies four distinct competitive regimes, while in 2D the structure collapses into a continuous multimodal density structure. This indicates that the separation observed at higher resolution is not preserved under coarse-graining. The regimes detected in the full space correspond to finer-scale structural variations embedded within a broader macroscopic organization. The verified concentration of leagues in preferred regions of the macro state-space indicates there are structural constraints governing competitive organization. As such, the contour topology provides a geometric representation of the common macroscopic representation underlying that league organization.

The study used 200 bootstrap replicates to evaluate the robustness of the clustering results. In each of these replicates, hierarchical clustering and DBSCAN were carried out using the same methods and parameter settings as in the main analysis. The degree of agreement between each bootstrap clustering and the reference clustering was measured using the adjusted Rand index (ARI). To assess the sensitivity of the DBSCAN solution to the neighborhood radius, we varied ε around the reference value $\varepsilon_{0}=1.5\times \mathrm{median}\left(d_{3\mathrm{NN}}\right)$, where $d_{3\mathrm{NN}}$ is the third-nearest-neighbor distance in the standardized macro state-space.

**Figure 24:**
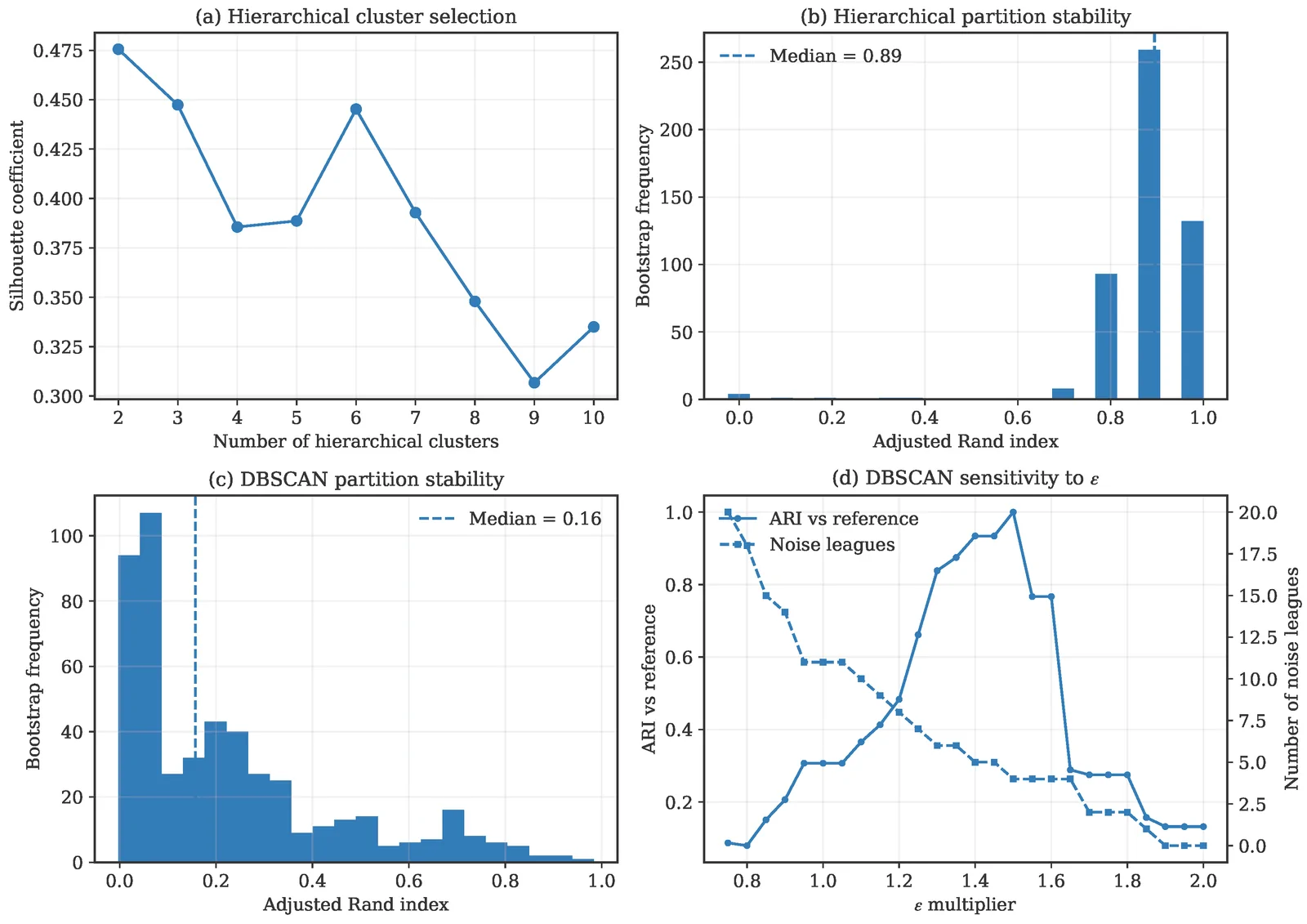
Bootstrap robustness of the hierarchical clustering and DBSCAN solution.

Figure 24 summarizes the bootstrap distributions of the clustering statistics, whereas Table 4 reports the corresponding numerical results. Hierarchical clustering exhibited high stability, with a median bootstrap ARI of 0.895 and a 95th percentile interval of 0.701–1.000, indicating that the two-cluster partition was reproduced consistently across bootstrap samples. In contrast, the DBSCAN solution was considerably more sensitive to bootstrap perturbations, with a median ARI of 0.157 and a substantially wider CI. This behavior is expected for density-based clustering, where small changes in the local point density may alter cluster boundaries and the identification of noise observations. As a result, the hierarchical clustering serves as the main evidence for the robustness of the macrostate partition, while the DBSCAN analysis must be seen as a supplementary exploratory evaluation of density-based structure.

The above analyses indicate that the seasonal macro state data are well approximated by a low-dimensional representation. The repeated appearance of similar structures across different methods such as dimensionality reduction, clustering, and distance-based techniques strongly suggests that the organization we see is a real feature of the system, not just a result of the selected methods.

From the identification of structures, clusters, regimes, and persistent geometric neighborhoods, the idea of common macroscopic organization emerges. A macroscopic organization class consists of leagues that occupy similar regions of the league-level macro state-space; as such, these leagues show similar macroscopic organization even if there are significant differences in microscopic details, such as match-level randomness or team-level composition. The existence of these classes suggests that the large-scale behavior of soccer competitions is influenced by common structural patterns that go beyond individual leagues.

The achieve results support the interpretation that (i) soccer leagues are complex adaptive, open, and non-equilibrium systems; (ii) their macroscopic organization is constrained to a low-dimensional space; and (iii) their long-term evolution exhibits signatures of shared organizational principles.

## 6. Conclusions

We have proposed a simple framework to study the long-term structure changes in soccer leagues. This framework is inspired by ideas from non-equilibrium statistical physics. Using a Bradley–Terry interaction model and latent-strength inference, we have demonstrated that league evolution shows (i) ongoing imbalances in competition, (ii) the rise of hierarchy among teams, and (iii) recurring structure patterns across various competitions. The macro state representations show basic geometric organization, clear clustering patterns, and similar structure relationships across leagues. These findings suggest shared principles that drive competitive league dynamics. They support the idea that soccer leagues are evolving complex systems in which broader organization comes from repeated team interactions rather than just league-specific details. This framework aims to provide a broad overview of league evolution instead of a detailed statistical-mechanical model. The Bradley–Terry approach that we use takes drawn matches into account. This approximation is intended for season-level latent-strength inference rather than for probabilistic modeling of draw outcomes. Future work should explore different latent-strength inference models such as Elo, Glicko, and Davidson types. Other future directions could include adding uncertainty quantification for the inferred latent strengths, creating specific models for temporal changes, and connecting macro state dynamics to external factors such as financial and structural limits.

## Figures and Tables

**Table 1:** Summary statistics of 38 soccer leagues.

League	Number of Seasons	Total Teams	Persistent Teams	Number of Matches	Average Goals Per Match
Argentina_Primera	15	43	9	5981	2.234
Austria_Bundesliga	13	20	5	2446	2.922
Belgium_Jupiler	31	40	4	8400	2.875
Brazil_SerieA	14	37	5	5319	2.387
China_SuperLeague	12	43	4	2808	2.910
Denmark_Superliga	13	20	5	2760	2.777
England_Championship	33	70	0	17,800	2.570
England_Conference	21	97	0	11,234	2.715
England_League1	33	91	0	17,639	2.597
England_League2	33	80	0	17,515	2.549
England_Premier	33	51	6	12,614	2.696
Finland_Veikkausliiga	14	23	4	2544	2.642
France_Ligue1	33	46	2	11,500	2.475
France_Ligue2	29	72	0	10,652	2.337
Germany_Bundesliga1	33	46	3	9970	2.945
Germany_Bundesliga2	32	82	0	9668	2.762
Greece_Ethniki	27	54	3	6995	2.458
Ireland_Premier	14	23	5	2563	2.568
Italy_SerieA	33	53	4	11,534	2.657
Italy_SerieB	29	85	0	11,441	2.419
Japan_JLeague	14	32	6	4523	2.616
Mexico_LigaMX	13	25	14	4319	2.644
Netherlands_Eredivisie	33	43	5	9736	3.070
Norway_Eliteserien	14	31	4	3382	2.992
Poland_Ekstraklasa	13	35	6	3776	2.639
Portugal_Liga1	33	70	0	9388	2.528
Romania_Liga1	13	40	3	3861	2.399
Russia_Premier	13	38	7	3160	2.522
Scotland_Championship	33	55	0	5935	2.662
Scotland_League1	29	36	0	5008	2.924
Scotland_League2	29	31	0	4921	2.861
Scotland_Premier	32	20	3	6688	2.680
Spain_LaLiga	33	49	4	12,592	2.662
Spain_Segunda	33	99	0	14,398	2.367
Sweden_Allsvenskan	14	35	7	3384	2.796
Switzerland_SuperLeague	13	18	4	2447	2.976
Turkey_Ligi1	32	65	4	9900	2.794
USA_MLS	14	31	18	5816	2.893

**Table 2:** Median bootstrap standard errors and 95% CI widths of the seasonal macroscopic observables across all league seasons.

Observable	Median Bootstrap Standard Error	Median 95% CI Width	Mean 95% CI Width
$\sigma_{t}^{2}$	0.1048	0.4037	0.4460
$G_{t}$	0.0467	0.1814	0.1846
$H_{t}^{out}$	0.0166	0.0641	0.0654
$H_{t}^{struct}$	0.0651	0.2477	0.2955
$CR_{t}^{4}$	0.0431	0.1663	0.1664
$HA_{t}$	0.0474	0.1832	0.1908

**Table 3:** Bootstrap and parallel-analysis validation statistics for the seasonal macrostate PCA. Bootstrap CIs were obtained from 500 replicates, and Horn’s parallel analysis used 1000 permutation datasets.

PC	Original Explained Variance Ratio	Bootstrap Mean Explained Variance Ratio	Bootstrap Standard Error	95% CI Low	95% CI High	Bootstrap Bias	Median Loading Similarity	Observed Eigenvalue	Permutation 95%	Retained
1	0.699	0.684	0.009	0.665	0.701	−0.015	0.992	4.200	1.156	Yes
2	0.175	0.186	0.008	0.173	0.202	0.011	0.964	1.052	1.090	No
3	0.106	0.099	0.004	0.093	0.107	−0.007	0.962	0.638	1.043	No
4	0.013	0.018	0.001	0.016	0.020	0.005	0.988	0.077	1.005	No
5	0.005	0.010	0.001	0.008	0.012	0.005	0.989	0.030	0.970	No
6	0.002	0.002	0.000	0.002	0.002	0.000	0.993	0.010	0.931	No

**Table 4:** Bootstrap validation statistics for the hierarchical clustering and DBSCAN analyses.

Method	Number of Groups	Median Bootstrap ARI	95% CI	Noise Observations
Hierarchical	2	0.895	0.701–1.000	–
DBSCAN	4	0.157	0.000–0.771	4

## Data Availability

The raw data supporting the conclusions of this article will be made available by the authors on request.
